# Wastewater-associated plastispheres: A hidden habitat for microbial pathogens?

**DOI:** 10.1371/journal.pone.0312157

**Published:** 2024-11-06

**Authors:** Ingun Lund Witsø, Adelle Basson, Marina Aspholm, Yngvild Wasteson, Mette Myrmel

**Affiliations:** 1 Faculty of Veterinary Medicine, Food Safety Unit, Norwegian University of Life Sciences, Ås, Norway; 2 Faculty of Veterinary Medicine, Virology Unit, Norwegian University of Life Sciences, Ås, Norway; University of Rome Tor Vergata: Universita degli Studi di Roma Tor Vergata, ITALY

## Abstract

Wastewater treatment plants (WWTPs) receive wastewater from various sources. Despite wastewater treatment aiming to remove contaminants, microplastics persist. Plastic surfaces are quickly colonized by microbial biofilm (“plastispheres”). Plastisphere communities are suggested to promote the spread and survival of potential human pathogens, suggesting that the transfer of plastispheres from wastewater to the environment could pose a risk to human and environmental health. The study aimed to identify pathogens in wastewater plastispheres, specifically food-borne pathogens, in addition to characterizing the taxonomic diversity and composition of the wastewater plastispheres. Plastispheres that accumulated on polypropylene (PP), polyvinyl chloride (PVC), and high-density polyethylene propylene (HDPE) surfaces exposed to raw and treated wastewater were analyzed via cultivation methods, quantitative reverse transcription PCR (RT‒qPCR) and 16S rRNA amplicon sequencing. RT‒qPCR revealed the presence of potential foodborne pathogenic bacteria and viruses, such as *Listeria monocytogenes*, *Escherichia coli*, norovirus, and adenovirus. Viable isolates of the emerging pathogenic species *Klebsiella pneumoniae* and *Acinetobacter* spp. were identified in the plastispheres from raw and treated wastewater, indicating that potential pathogenic bacteria might survive in the plastispheres during the wastewater treatment. These findings underscore the potential of plastispheres to harbor and disseminate pathogenic species, posing challenges to water reuse initiatives. The taxonomic diversity and composition of the plastispheres, as explored through 16S rRNA amplicon sequencing, were significantly influenced by the wastewater environment and the duration of time the plastic spent in the wastewater. In contrast, the specific plastic material did not influence the bacterial composition, while the bacterial diversity was affected. Without efficient wastewater treatment and proper plastic waste management, wastewater could act as a source of transferring plastic-associated pathogens into the food chain and possibly pose a threat to human health. Continued research and innovation are essential to improve the removal of microplastics and associated pathogenic microorganisms in wastewater.

## Introduction

During the past few decades, the production of plastic has been constantly increasing [1]. The high strength, durability, corrosion resistance, and low cost, are among the many characteristics of polymers that make plastic a competitive product in the consumer market [2]. Approximately 6300 million metric tons of plastic have been manufactured since 2015, 76% of which have been discarded and are accumulating in landfills and environments [3]. The most common plastic produced is polyethylene (PE, 36%), followed by polypropylene (PP, 21%) and polyvinyl chloride (PVC, 12%). Plastic packaging is mostly made of PE and PP, whereas up to 69% of PVC is used in the building and construction industry. An increase in plastic production is followed by an increase in the accumulation of plastic in the environment [4]. In addition to the increasing challenges related to plastic pollution and its impact on ecosystems, plastic pollution has also become a concern regarding food safety. Plastic fragments and particles < 5 mm are referred to as microplastics (MPs). They are considered significant food contaminants because of their chemical pollutants and additives that are recognized as toxic to humans and are now controlled by the European Commission`s Rapid Alert System for Food and Feed (RASFF) and the European Food Safety Authority (EFSA) [5, 6].

Wastewater treatment plants (WWTPs) receive wastewater from households, institutions, industries, and rainwater runoff [7]. Municipal WWTPs are recipients of plastic particles of different sizes originating from household activities such as washing synthetic clothes, personal care, and rinsing cosmetic products directly down household drains [8]. In addition, tire wear particles represent a major source of microplastics entering the environment [9] and are often transported into WWTPs through storms and meltwater [10, 11]. When entering treatment plants, wastewater is generally subjected to physical, biological, and chemical processes to remove solids, organic matter, and pathogens before discharge. Treatment processes reduce pollutant loads to ensure environmental safety [12, 13]. Although large and small plastic pieces in influent wastewater are removed through filtering, coagulation, and sedimentation processes, no wastewater treatment technique results in complete plastic retention [14]. Consequently, MP has been found in wastewater influent, effluent, and sludge, implying that wastewater is a source of MP released into the environment [7, 15, 16]. Additionally, potentially pathogenic bacteria, such as *Klebsiella pneumonia*, *Acinetobacter baumannii*, and *Enterobacteriaceae* spp, as well as viruses like norovirus and adenovirus, have been found in wastewater effluent [17–19]. This indicates that pathogens can bypass the treatment process and be released into the environment.

Wastewater contains a “core microbiome”, including microorganisms such as *Comamonas*, *Pseudomonas*, *Acidovorax*, and *Arcobacter*, which play a role during the bioremediation process. Additionally, wastewater may contain waterborne microorganisms such as *Legionella*, *Vibrio*, and *Leptospira*, as well as environmental microorganisms like *Acinetobacter*, *Aeromonas*, and *Pseudomonas*. Furthermore, fecal bacteria such as *Campylobacter*, *Clostridium*, *Salmonella*, and *Shigella* as well as enteric viruses of human and animal origin like adenovirus, norovirus, and enterovirus, are found in the wastewater [18, 20–22]. The high concentrations of microorganisms in WWTPs provide an ideal environment for biofilm formation on plastic surfaces passing through the treatment steps. Plastic litter offers a durable substrate for the growth of microbial biofilm ecosystems referred to as “plastispheres” [23]. The composition of these ecosystems can differ substantially from that of microbial communities in the surrounding environment [23–25]. Biofilms contain a matrix of extracellular polymeric substances (EPSs) that provide an advantage in terms of competitiveness and survival. This matrix supports nutrient acquisition and shields against external pressures such as disinfectants, radiation, and shear forces [26, 27]. Most assessments of plastispheres have been done by sequencing technologies [28], thus there is a knowledge gap in detecting pathogens by using a combination of molecular techniques and culturing to identify viable and potentially virulent strains.

Enteric viruses such as adenovirus (AdV) and norovirus (NoV) have been detected in all steps of the wastewater treatment process [29, 30]. While studies have discovered viruses in freshwater plastispheres [31, 32], and both naked plastics and biofilms on PE from treated wastewater have been shown to attract these enteric viruses [33], the presence of pathogenic viruses in wastewater plastispheres has not been thoroughly explored. Despite some studies emphasizing the role of plastispheres as carriers of pathogenic bacteria in wastewater [34, 35], the presence of pathogenic viruses in wastewater plastispheres has not been given much attention. In addition to contributing to increased knowledge regarding pathogenic bacteria present in wastewater plastispheres, our study aims to address this knowledge gap to improve the understanding of the potential transmission of food-borne pathogens through wastewater treatment plants.

Moreover, the identification of human pathogens in plastispheres across various stages of wastewater treatment implies that the plastisphere could serve as a durable shelter for pathogens [34]. The persistence of microorganisms and the longevity of plastics makes them ideal transport vectors of pathogens from highly polluted environments (such as wastewater) to natural environments. Consequently, plastispheres could play a role in the spread of pathogenic species in the environment. Treated wastewater is an alternative water source for irrigation, especially in regions facing water scarcity [12]. However, as irrigation water is recognized as a source of microplastics in soil used for crop production [36], and treated wastewater is associated with a high load of pathogens, using treated wastewater for irrigation has raised concern. Altogether, this implies that the potential for plastic-associated pathogens to enter the agricultural systems and food systems is high [37]. There is a need to increase awareness of the presence and associated risk of pathogens entering the food systems via microplastics. The consequences and long-term effects of plastic pollution and exposure to microplastics in different environments are undergoing intense research focus [38]. Recently, the attention has shifted to the potential risk to human and environmental health posed by the presence and exposure to plastic colonized by foodborne pathogens. This study aims to increase the knowledge about plastic surfaces as a source of potential pathogenic microorganisms in wastewater, emphasizing the need for proper and improved plastic waste handling and wastewater management.

The present study sampled plastic-associated biofilms from raw and treated wastewater from a WWTP. The main objective of this study was to identify pathogens in plastispheres, with a specific focus on food-borne pathogens, using RT‒qPCR, 16S rRNA amplicon sequencing, and cultivation-dependent methods, followed by identification via MALDI‒TOF. The taxonomic diversity and composition of the collected plastispheres were analyzed to describe the microbiomes obtained from raw and treated wastewater under the influence of plastic surfaces and the duration of time spent in distinct wastewater habitats.

## Materials and methods

### WWTP sampling

VEAS is Norway’s largest WWTP. It receives municipal wastewater from a population of 870,000 in both the Oslo and Viken districts. The plant treats 100–110 million m^3^ of municipal wastewater annually, which includes sewage from five major hospitals in the Oslo region [39]. VEAS receives up to 11,000 L/sec of sewage and wastewater daily. Wastewater undergoes several treatment steps that transform the raw sewage into effluent, which is released into the environment [39].

When the wastewater and sewage enter VEAS, a mesh screen separates larger solid objects from the water. Next, a grit removal chamber was used to remove stones, gravel, and sand. Following this, the water is transported into large basins where the primary treatment process starts. At this stage, chemicals are added to remove phosphorus and organic matter by aggregating smaller particles, which then sediment into sludge. The sludge is pumped out from the bottom of the pool, while the water is subjected to biological purification (secondary treatment). At this stage, microorganisms in the wastewater microbiome remove carbon, nitrogen, and phosphorus, and the number of pathogens is reduced in this step. The wastewater effluent from VEAS is discharged into Oslo fjord after secondary treatment, where the salinity of the ocean water aids in further cleaning. The whole treatment process lasted approximately three to five hours [39].

The plastic types used in this study as a matrix for biofilm formation were PP, PVC, and HDPE (high-density polyethylene), as these are considered one of the main common plastic materials found as plastic pollution in the environment [40], and are representative of what is commonly found in WWTP [11, 14, 41]. Plastic pieces, 4 × 6 × 0.06 cm^3^ in size, were mounted to a rope with a weight attached to the end to submerge them vertically in wastewater. The pieces were surface sterilized with hypochlorite before being lowered into the basins. The chosen sampling locations were a pool with a continuous flow of raw wastewater and a basin containing effluent wastewater from the final stage of treatment (named “treated wastewater”).

The experiment took place in August and September 2021. Even though the time MPs spend in the WWTP might be short, the plastispheres were allowed to grow for 14 days and 30 days (hereafter designated D14 and D30, respectively) in both environments (raw and treated wastewater, respectively). This was based on a previous pilot study, where the amount of biofilm formed on the plastic pieces after a shorter time was insufficient to extract enough high-quality DNA. After harvesting, the plastic pieces were placed in sterile containers containing PBS and transported to the laboratory within two hours. Pictures of the biofilm formed on the different plastic pieces can be found in the supplementary (S1 Fig). The pieces were rinsed carefully three times with PBS to remove loosely attached organic material and were immediately analyzed for the presence of pathogenic bacteria by cultivation or frozen at -80°C for later extraction of DNA and RNA.

### Isolation and identification of potential bacterial pathogens from wastewater biofilms

Potential pathogenic bacteria from the wastewater biofilms were isolated and identified as described previously [31]. Briefly, one surface of the plastic pieces was swabbed and spread onto agar plates (blood agar and LB agar). The plates were incubated at different conditions (aerobic, or anaerobic, at 37°C or 22°C). Single colonies with unique morphologies were purified in two rounds of subculturing under the described conditions (S1 Table). Moreover, the other surface of each piece was divided into five similar-sized areas. Each area was swabbed and streaked onto selective media for *Aeromonas* spp., *Campylobacter* spp., *Escherichia coli*, *Listeria* spp., and *Salmonella* spp. and incubated under the conditions listed in S1 Table. Stocks of all bacterial isolates were stored at -80°C until further characterization. The acquired isolates were identified by MALDI-TOF as described previously [31].

### Extraction of DNA/RNA from biofilms

Triplicates of each plastic type were pooled and regarded as a single sample. Three replicates for each sample were included in the study. DNA was extracted from the raw and treated wastewater samples collected after two and four weeks (S2 Table).

The samples were thawed on ice, and the biofilms from both sides of the three plastic pieces were pooled by scraping off the surface into a ZR BashingBead Lysis tube with 0.1- and 0.5-mm beads containing 750 μL of DNA/RNA Shield^TM^ provided in the extraction kit (ZymoBIOMICS DNA/RNA Minprep kit, Nordic BioSites AS, Norway). The samples were homogenized using MP Bio FastPrep-24 (VWR) at 6 m/s for 5 × 45 s, with 15 s breaks between each cycle while kept on ice. After bead beating, DNA and RNA were extracted separately using the ZymoBIOMICS DNA/RNA Miniprep Kit according to the manufacturer’s instructions and as described previously [31]. The DNA was stored at -80°C until use. The extracted DNA was used for the detection of specific pathogens by qPCR 16S rRNA gene sequencing as described below. The isolated RNA was screened for NoV via RT‒qPCR as described below.

### RT‒qPCR for detection of pathogens

As 16S rRNA amplicon sequencing cannot be used to characterize bacteria at the species level or detect viruses, RT-qPCR was performed to assess the presence of well-known foodborne pathogenic bacterial species and foodborne viruses, as previously described [31]. Briefly, primers (Thermo Fisher) targeting VS1, *tir*, and *hlyA* were used for the detection of *Campylobacter jejuni*, enteropathogenic *Escherichia coli* (EPEC), and *Listeria monocytogenes*, respectively, using the AriaMx Real-Time qPCR System (S3 Table) [42–44].

One-step TaqMan RT‒qPCR was used to detect adenovirus 40/41 (AdV) and norovirus GI/GII (NoV). An AriaMx Real-time PCR system (Agilent Technologies, Santa Clara, California, USA) was used for virus detection and data analysis [31]. All primers- and probe sequences and concentrations are given in the S3 Table. Positive and negative controls were used in the RT-pPCR assays to ensure the validity of the results. Known quantities of target pathogen representatives for each assay were used as positive controls. Nuclease-free H_2_O was used as a negative control in each assay to ensure no contamination or false positives.

### Amplification, quantification, and sequencing of 16S rRNA gene

The bacterial compositions of the plastispheres were characterized by amplicon sequencing of the V3-V4 region of the 16S rRNA gene, which was conducted at Novogene Genome Sequencing Company (Company Limited, Cambridge UK). Briefly, amplicons were generated with the primers 515F and 806R connected with barcodes. PCR was conducted using Phusion^®^ High-Fidelity PCR Master Mix (New England Biolabs, Ipswich, MA, USA), and products of the proper size (400–450 bp) were selected for 2% agarose gel electrophoresis. Sequencing libraries were generated using the NEBNext® Ultra DNA Library Pre Kit for Illumina following the manufacturer`s recommendations, and index codes were added. Library quality was assessed on a Qubit@ 2.0 fluorometer (Thermo Scientific) and an Agilent Bioanalyzer 2100 system. Finally, the library was sequenced on an Illumina platform, generating 250 bp paired-end reads.

### Data processing and analyses

Data processing was conducted by Novogene Genome Sequencing Company. Briefly, quality filtering of the raw reads was performed under specific filtering conditions to obtain high-quality clean reads according to the Cutadapt quality control process (V1.9.1, http://cutadapt.readthedocs.io/en/stable/) [45]. Paired-end reads were assigned to samples based on their unique barcodes and were truncated by removing the barcode and primer sequences. Paired-end reads were merged using FLASH (V1.2.7) (http://ccb.jhu.edu/software/FLASH/) [46], and the resulting splicing sequences were called raw tags. Quality filtering of the raw tags was performed to obtain high-quality clean tags [47] according to the QIIME quality control process (V1.7.0) (http://qiime.org/scripts/split_libraries_fastq.html) [48]. The tags were compared with the reference database (SILVA138 database) using the UCHIME algorithm (UCHIME Algorithm) [49] to detect chimeric sequences (https://drive5.com/usearch/manual/chimeras.html). Finally, the chimeric sequences were removed, and effective tags were obtained [50].

Sequence analysis was performed by Uparse software (Uparse v7.0.1001) using all the effective tags [51]. Sequences with ≥ 97% similarity were assigned to the same OTUs. The representative sequence for each OTU was screened for further annotation. The use of QIIME (version 1.7.0) [52] in the Mothur method was performed against the SSUrRNA database of the SILVA138 Database for species annotation at each taxonomic rank (threshold: 0.8~1) (kingdom, phylum, class, order, family, genus, species) [53, 54]. In 2021, Oren and Garrity presented name changes for all bacterial phyla [55]. The SILVA138 database was not updated with the names of the new phyla at the time of the study; thus, the old names were used in the analysis.

### Statistical analysis

OTU abundance information was normalized using a standard sequence number corresponding to the sample with the fewest sequences. Subsequent alpha- and beta-diversity analyses were performed based on these normalized data. The diversity was analyzed with “Plastic”, “Environment” and “Days” as possible associated variables. Alpha diversity is applied to analyze the complexity of biodiversity for a sample through two indices: Chao1 and Shannon diversity. These indices were calculated with QIIME (version 1.7.0) and displayed in plots using the package ggplot2 (version 3.5.1) in RStudio (version 2023.06.2 +561) [56–58]. To investigate the diversity indices, two analysis of variance (ANOVA) models with interaction effects were constructed with the two different alpha measures as response variables and the three possible associated variables as explanatory variables. Tukey’s post hoc test was performed for pairwise comparisons of the results. The statistical analysis of the alpha diversity was performed in RStudio (version 2023.06.2 +561). For all the statistical analyses, the model assumptions were checked and fulfilled, and p < 0.05 was considered to indicate statistical significance.

Beta diversity analysis was used to evaluate differences in species complexity among the samples. Beta diversity on weighted UniFrac was calculated by QIIME software (Version 1.7.0) [56]. A distance matrix of weighted UniFrac was transformed to a new set of orthogonal axes in a principal coordinate analysis (PCoA), by which the first principal coordinate demonstrated the maximum variation factor, the second principal coordinates demonstrated the second maximum variation factor, and so on. PCoA was performed with the WGCNA package (version 1.73) and ggplot2 package (version 3.5.1) in R software (version 2.15.3) [58, 59].

To analyze the influence of the different variables (plastic, environment, and days) on the bacterial community composition, permutational multivariate analysis of variance (PERMANOVA) with three-way interaction effects was performed using the weighted UniFrac distance. All PERMANOVAs were performed in RStudio (version 2023.06.2 +561) using the Vegan package (version 2.6.8) with the adonis() function [58, 60].

The statistical analysis revealed no significant difference in the diversity or composition between the plastic materials, so the relative abundance of each plastic surface was combined to determine the relative abundance of the pathogenic genera present in the plastispheres.

For a more detailed investigation of the bacterial diversity of the taxa, relative abundances were plotted for the 20 most abundant phyla and the 20 most abundant genera using the ggplot2 package in RStudio (version 2023.06.2 +561). A visual examination of the plots together with the relative abundance was performed.

A network analysis was performed to explore the inter-associations among the different taxa within the plastisphere communities [61]. The pairwise Spearman`s correlation coefficient (ρ) was calculated, and a matrix was constructed to investigate the potential relationship in the plastisphere communities. Statistically significant correlations between nodes were defined as ρ ≥ 0.8 with a p-value of ≤ 0.01. Nodes and edges in the network represent OTUs at the phylum level. The analysis results were visualized using the RStudio (version 2024.04.2).

## Results

### Detection of pathogens

The results from the three different plastic surfaces were combined to determine the relative abundance of pathogenic bacteria in the plastispheres. According to the 16S rRNA sequence analysis, the plastispheres comprised the genera *Salmonella*, *E*. *coli/Shigella*, *Listeria*, and *Campylobacter*, all recognized for containing foodborne pathogen variants within their genus. Although their abundance was low (< 1%), these genera were detected after 14 days (D14) and 30 days (D30) in the raw or treated wastewater (Table 1).

**Table 1:** The abundance (%) of genera harboring food-borne pathogens.

	Raw wastewater		Treated wastewater	
	D14	D30	D14	D30
*Salmonella*	0.655%	0.751%	0.082%	0.076%
*Shigella/E*. *coli*	0.333%	0.627%	0.122%	0.069%
*Listeria* spp.	0.137%	0.227%	0.025%	0.038%
*Campylobacter* spp.	0.021%	0.013%	0.001%	0.000%
*Bacillus* spp.	3.510%	0.582%	0.026%	0.034%
*Pseudomonas* spp.	1.224%	0.672%	0.022%	0.033%
*Acinetobacter* spp.	7.621%	12.649%	0.115%	0.080%
*Providencia* spp.	0.001%	0.000%	0.007%	0.009%
*Serratia* spp.	0.013%	0.003%	0.000%	0.001%
*Yersinia* spp.	0.004%	0.004%	0.018%	0.023%

The relative abundance of genera recognized for harboring food-borne pathogens was assessed in plastispheres submerged in wastewater for 14 or 30 days. respectively. The results include the abundance (%) of each genus on the three different plastic surfaces combined (n = 9).

Furthermore, 16S rRNA sequences attributed to potential opportunistic pathogens such as *Klebsiella pneumoniae*, *Aeromonas hydrophila*, *Serratia marcescens*, and *Enterobacter* spp. were also detected in the wastewater plastispheres. *Acinetobacter* spp. was one of the most abundant taxa found in the plastispheres from raw wastewater and increased in abundance from D14 to D30 (from 7% to 12%). On average its relative abundance in treated wastewater plastispheres was 0.095% (Table 1). *K*. *pneumoniae* and *Acinetobacter* spp. were cultivated from plastispheres and identified by MALDI-TOF from raw and treated wastewater.

Accordingly, potentially pathogenic *E*. *coli* and *L*. *monocytogenes* were confirmed by RT‒qPCR in the raw and treated wastewater plastispheres (Table 2). None of the abovementioned potential bacterial foodborne pathogens were successfully isolated through cultivation.

**Table 2:** Pathogenic viruses and bacteria were detected by (RT)-qPCR in the plastispheres.

	Raw wastewater		Treated wastewater	
Pathogen	D14	D30	D14	D30
*C*. *jejuni*	0/6	0/6	0/6	0/6
*L*. *monocytogenes*	**1/6**	**1/6**	0/6	**3/6**
EPEC	**6/6**	**6/6**	**6/6**	**6/6**
Norovirus GI	0/6	**5/6**	0/6	**1/6**
Norovirus GII	**4/6**	**6/6**	**5/6**	**5 /6**
Adenovirus	**6/6**	**6/6**	**6/6**	**6/6**

Pathogenic viruses and bacteria were detected by (RT)-qPCR in plastispheres submerged in raw or treated wastewater for 14 or 30 days. respectively. A total of 12 samples (6 samples for D14 and 6 for D30) were analyzed for each environment. The results are expressed as the ratio of positive samples to the total number of samples analyzed. The positive results are highlighted in bold letters.

AdV and NoV GII were detected at D14 and D30 in the raw and treated wastewater, while NoV GI was detected only in the plastispheres from D30 (Table 2).

### Bacterial community composition and diversity analysis

#### Bacterial community composition

After quality filtering and chimera removal, the numbers of effective reads (nochime reads) were 58,000 ± 5,400 (mean ± SD) in the raw wastewater and 52,900 ± 3,600 (mean ± SD) in the samples from the treated wastewater. An average of 2,220 OTUs were identified in the raw wastewater samples, compared to 1,256 OTUs in the treated wastewater samples.

**Fig 1:**
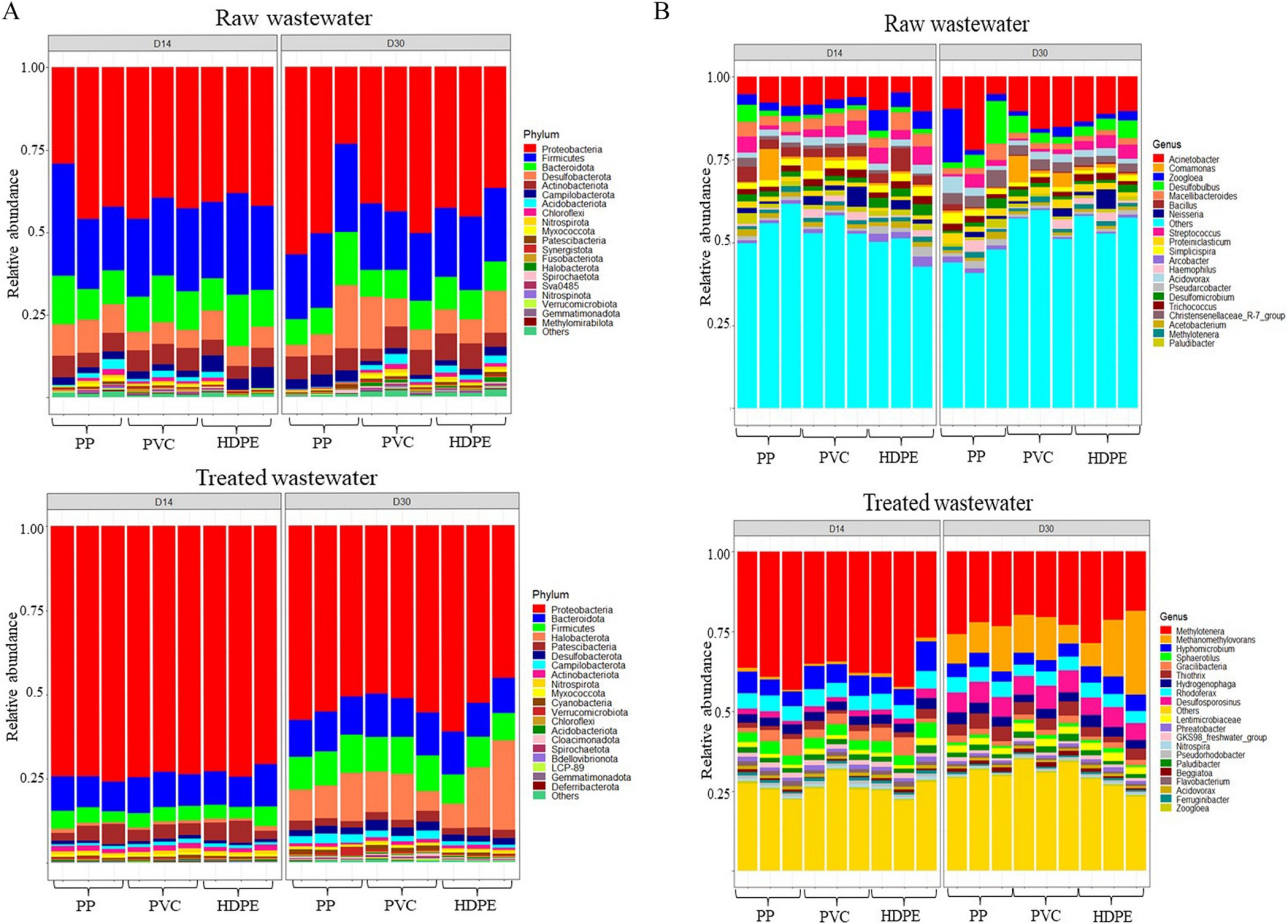
Dominant taxa in the wastewater plastisphere communities. The relative abundance of the 20 most abundant phyla (A) and genera (A) in the raw and treated wastewater plastispheres. Each color represents one phylum or genus, and the length of the patch represents the relative abundance of the phylum/genus. Each bar represents a group of three replicates. The different plastic materials are listed on the x-axis, and the y-axis denotes the abundance of the phyla and genera. The bar plots show the clear differences in the bacterial communities between raw wastewater plastispheres and treated wastewater plastispheres.

The phylum Proteobacteria dominated the plastisphere communities from raw wastewater (D14: mean = 40.72 ± 4.78%, D30: mean = 43.37 ± 8.97%), followed by Firmicutes (D14: mean = 25.18 ± 4.33, D30: mean = 21.42 ± 2.39%) and Bacteroidota (D14: mean = 11.84 ± 2.16%, D30: mean = 9.46 ± 2.41%) (Fig 1A and S4 Table). In contrast, the plastisphere communities from treated wastewater at D14 were dominated by Proteobacteria (mean = 73.76 ± 1.33%) and Bacteroidota (mean = 10.05 ± 1.04%). At D30, Proteobacteria (mean = 53.24 ± 4.42%), Bacteroidota (mean = 12.97 ± 5.92%), Firmicutes (mean = 11.65 ± 0.96%), and Halobacteriota (mean = 9.83 ± 0.97%) dominated the plastispheres (Fig 1A and S4 Table).

*Acinetobacter* was the most abundant genus in the plastispheres from the raw wastewater at both D14 and D30 (mean = 7.62 ± 1.92% and mean = 12.64 ± 4.50%, respectively) (S5 Table). Other abundant genera in plastispheres from raw wastewater at D14 were *Macellibacteriodes* (mean = 3.66 ± 0.79%), *Zoogloea* (mean = 3.65 ± 1.30%), *Bacillus* (mean = 3.50 ± 1.35%), and *Streptococcus* (mean = 3.33 ± 1.48%). Approximately 50% of the genera identified in plastispheres from raw wastewater were low-abundance taxa (Fig 1B).

*Methylotenera* was the most abundant genus in the plastispheres from treated wastewater at both D14 and D30 (mean = 37.09 ± 4.59%, mean = 22.59 ± 2.95%, respectively). Other abundant genera in treated wastewater plastispheres sampled at D14 were *Hyphomicrobium* (mean = 6.14 ±1.39%), *Rhodoferax* (mean = 4.78 ± 0.42%) and *Sphaerotilus* (mean = 3.42 ± 0.56%). At D30, *Methanomethylovora* (mean = 12.9 ± 5.88%), *Desulfosporosinus* (mean = 6.23 ± 0.59%), *Hyphomicrobium* (mean = 4.31 ± 0.75%), and *Rhodoferax* (mean = 4.32 ± 0.46%) were among the most abundant genera (Fig 1B, S5 Table).

### Bacterial community diversity and clustering

The Shannon diversity of the bacterial communities within the plastispheres was significantly influenced by all the variables (plastic material, environment, and days), as well as their interactions (Table 3).

**Table 3:** Alpha-diversity analysis of the plastispheres bacterial communities.

	Chao1		Shannon	
Variables/ Interactions	F- value	Pr (<F)	F- value	Pr (<F)
Plastic	1.019	0.376	4.797	**0.017**
Environment	120.592	**< 0.001**	437.206	**< 0.001**
Days	0.002	0.961	9.324	**0.005**
Plastic:Environment	1.011	0.378	0.545	0.587
Plastic: Days	9.283	**0.001**	4.440	**0.022**
Environment: Days	0.628	0.436	9.274	**0.005**
Plastic:Environment: Days	8.417	**0.002**	8.823	**0.001**

An investigation of the alpha diversity indices was conducted using ANOVA models with three-way interaction effects. The table shows the results from the three-way interaction model for Shannon and Chao1 as response variables. The two ANOVA models show that the wastewater environment influenced the alpha diversity of the plastispheres the most. The statistically significant interactions (p < 0.05) are marked with bold letters.

**Fig 2:**
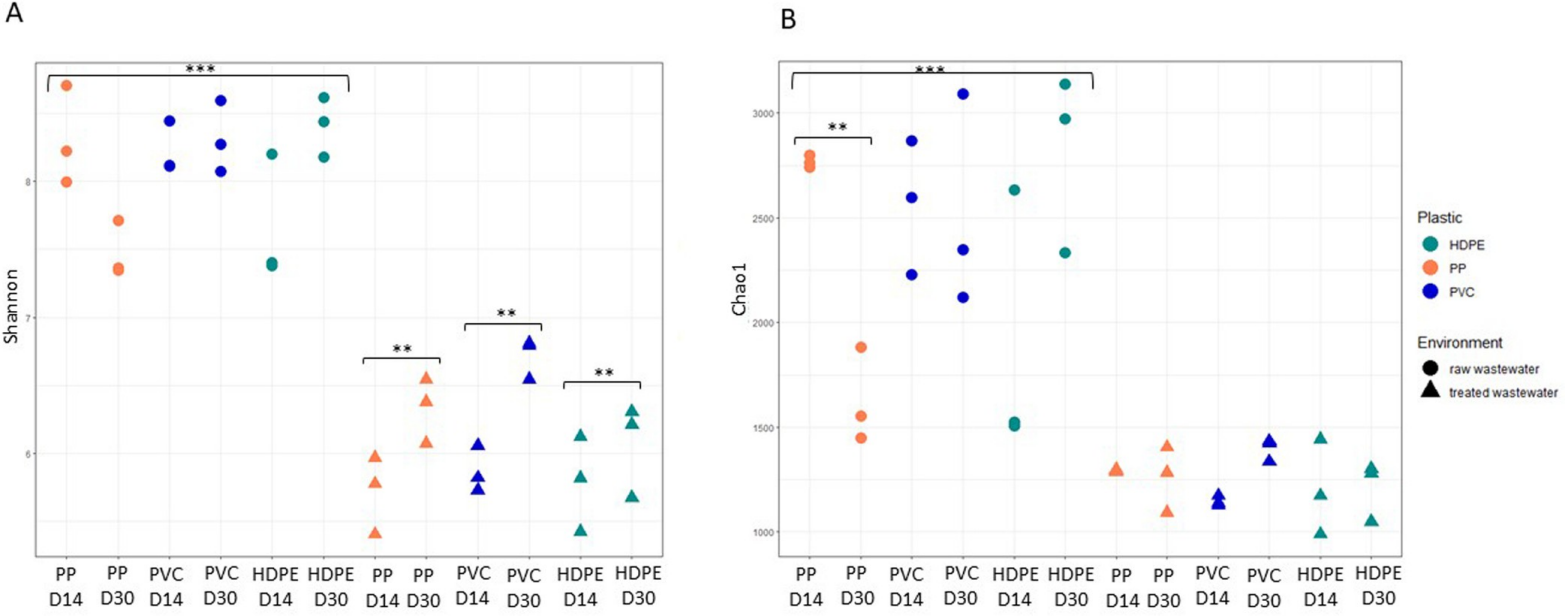
Bacterial alpha diversity in the wastewater plastispheres. The higher alpha diversity in the plastispheres from raw wastewater indicates more heterogenous and stable bacterial communities within this environment compared to the communities within treated wastewater plastispheres. The alpha diversity was calculated based on the OUT abundances from the bacterial communities on various plastic materials (PP, PVC, and HDPE) obtained from raw or treated wastewater during the experiment (D14 and D30). The x-axis represents the different samples in the study, while the y-axis denotes the values of the alpha diversity indices; (A) richness and evenness (Shannon index) and (B) richness (Chao1 index) of the microbiome. In cases with significant interactions according to three-way ANOVA, p-values for simple main effects from Tukey post hoc tests are presented. Asterisks represent statistical significance (* p < 0.05, ** p < 0.01, *** p < 0.001).

The post hoc Tukey test showed that plastispheres from the raw wastewater had greater Shannon diversity than those from the treated wastewater (diff: 1.987, 95% CI: [1.791; 2.183], p < 0.001). Among the plastispheres collected from raw wastewater, those from PVC and HDPE had greater Shannon diversity than did those collected from PP (Tukey test, PVC; diff = 0.299, 95% CI: [0.009; 0.590], p = 0.04; HDPE; diff = 0.323, 95% CI: [0.032; 0.614], p = 0.027). Overall, the Shannon diversity of the plastispheres from treated wastewater increased from D14 to D30 (diff = 0.290, 95% CI: [0.094; 0.486], p = 0.005), indicating the dynamic nature of the plastispheres. This difference was not observed for plastispheres that had been submerged in raw wastewater. These results are visualized in Fig 2A.

**Fig 3:**
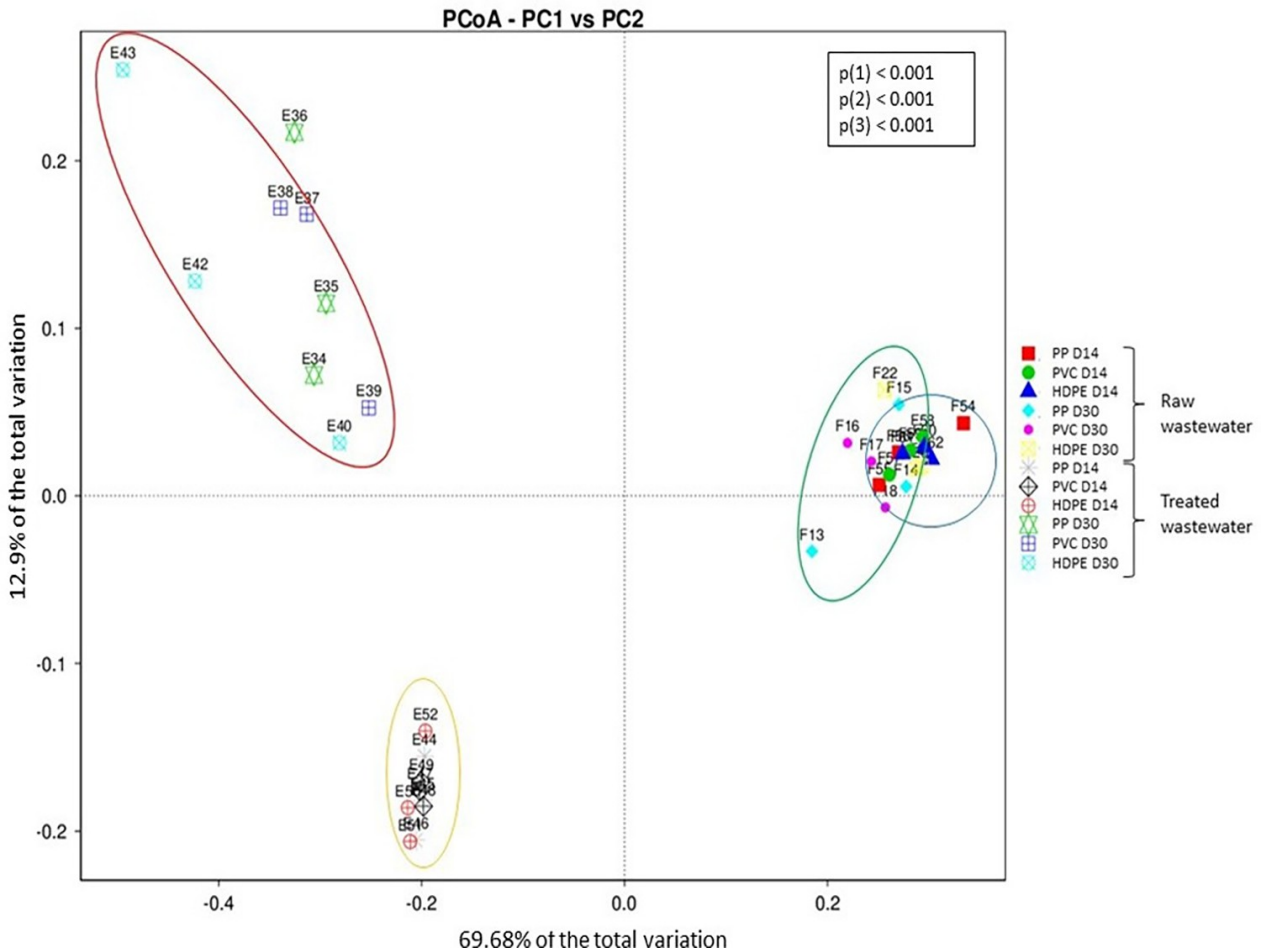
Composition differences in wastewater plastispheres. Principal coordinate analysis plot illustrating the weighted UniFrac distances between OTUs in the plastispheres from raw and treated wastewater. The colored circles represent different groups: blue (raw wastewater at D14), green (raw wastewater at D30), orange (treated wastewater at D14), and red (treated wastewater at D30). The plot highlights that most of the variation (68.68%) is attributed to environmental factors, highlighting the significant impact of the surrounding environment on microbial diversity in the plastispheres. Statistically significant p-values (p < 0.05) from PERMANOVA are provided for the variables and their interactions: (1) environment, (2) days, and (3) environment: days.

The species richness (Chao1) of the bacterial communities in the plastispheres was significantly affected by the environment but not by the type of plastic material or the time the plastic had been submerged in the wastewater (Table 3). There was a statistically significant interaction effect between the plastic material and days (F = 9.283, p < 0.001). The post hoc Tukey test showed that the plastispheres from the raw wastewater had greater richness than those from the treated wastewater (diff: 1113.178, 95% CI: [903.963; 1322.393], p < 0.001). Additionally, differences in richness were observed for the plastic types submerged in raw wastewater at D14 and D30. Specifically, plastispheres formed on PP had greater richness at D14 than at D30 (diff = -1139.047, 95% CI: [-2034.332; -243.762], p = 0.005), while in plastispheres on HDPE, the richness was greater at D30 than at D14 (diff = 926.441, 95% CI: [31.156; 1821.726], p = 0.038). There was no statistically significant difference in the richness of the plastispheres from treated wastewater. These results are summarized and visualized in Fig 2B. The results from the alpha diversity analysis reflect a more complex, heterogeneous, and robust microbial community in the raw wastewater plastispheres compared to the plastispheres from treated wastewater. These findings underscore the role of environmental factors in shaping the microbial diversity in plastispheres. Further, the beta diversity of the bacterial communities in the plastispheres revealed a significant difference in bacterial composition between the two environments and between days but not between plastic types (Table 4). PERMANOVA showed that the main source of variation among the samples was the environment from which they were collected (F = 154.842, p < 0.001) (Table 4), which was also supported by the PCoA plot (Fig 3).

**Table 4:** A PERMANOVA with three-way interaction effects using weighted UniFrac distance.

Variables/Interactions	R^2^	F- value	Pr(>F)
Plastic	0.017	1.344	0.246
Environment	0.674	154.842	**0.001**
Days	0.085	19.689	**0.001**
Plastic: Environment	0.018	1.401	0.238
Plastic: Days	0.021	1.632	0.171
Environment: Days	0.079	18.251	**0.001**
Plastic:Environment:Days	0.016	1.254	0.303

A PERMANOVA analysis with three-way interaction effects was conducted using weighted UniFrac distance to assess the impact of various variables on the beta diversity of the bacterial community composition. The wastewater environment, duration of exposure, and their interaction significantly influenced the beta diversity of the bacterial communities. The statistically significant (p > 0.05) interactions are indicated by bold letters.

Moreover, the plastispheres from treated wastewater formed distinct clusters based on the duration of exposure (days) to the wastewater (Fig 3). For the plastispheres from the raw wastewater, there was an overlap between D14 and D30. This was confirmed by the PERMANOVA, which indicated a significant interaction effect of environment and days (F = 18.251, p < 0.001) (Table 4), Altogether these results underscore the profound impact of environmental conditions on the microbial community structure of the plastispheres. The distinct clustering based on the duration of exposure suggests that the bacteria in the plastispheres adapt differently over time in varying environments.

**Fig 4:**
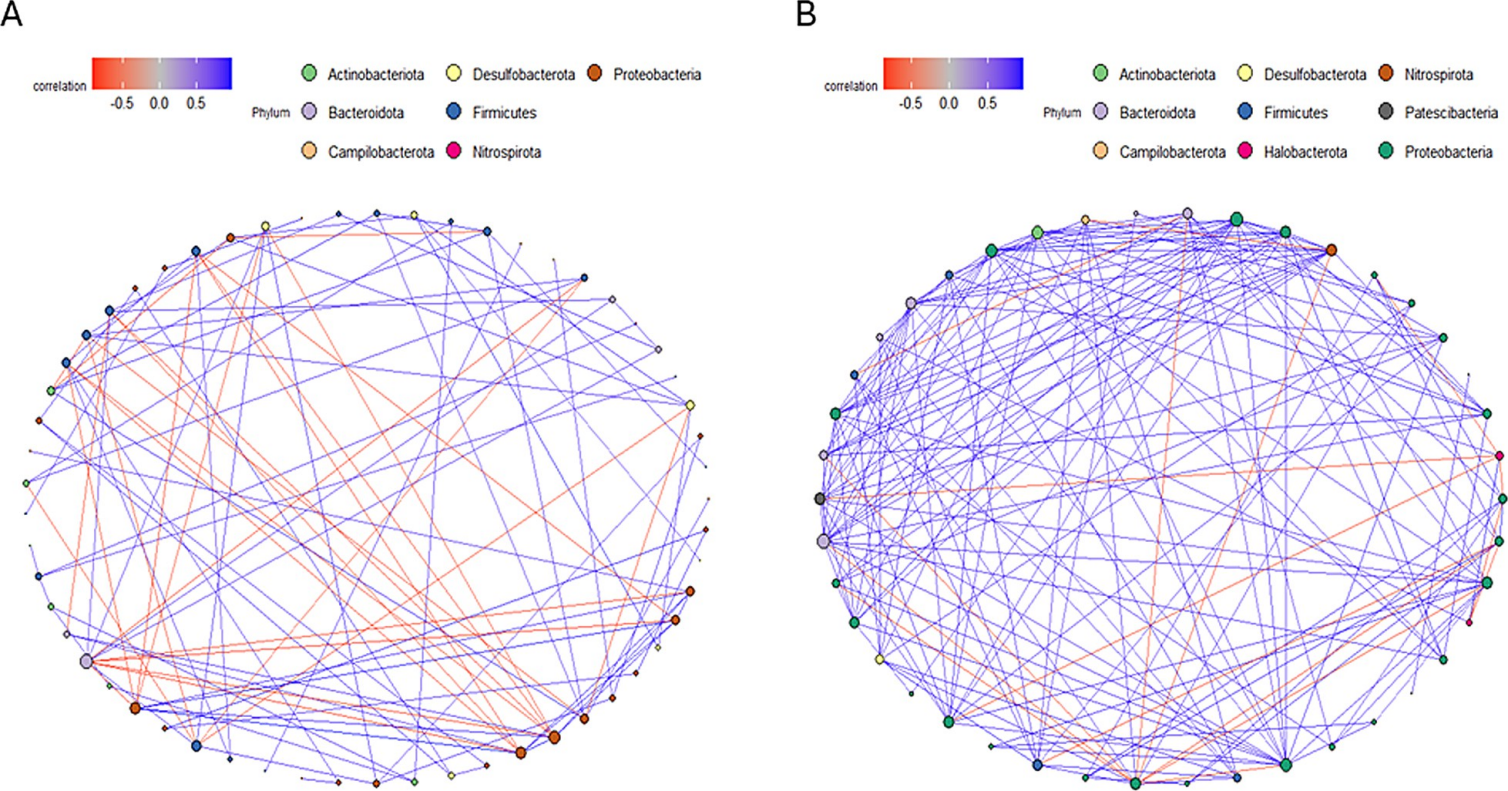
Co-occurrence patterns of bacterial taxa in raw (A) and treated (B) wastewater plastisphere. In this network analysis, bacterial OTUs are represented as nodes. The node colors correspond to distinct phyla, and the size of the nodes reflects phyla abundance. Blue edges indicate positive correlations, while red edges indicate negative correlations between nodes.

Due to the stronger effects of the environments on the bacterial communities in the plastispheres, a co-occurrence network analysis of the plastispheres from the two environments was performed. The co-occurrence network analysis indicates distinct interaction dynamics between the bacterial communities of the raw and treated wastewater plastispheres (Fig 4). The network from the raw wastewater plastispheres exhibited fewer edges and more negative correlations, while the network from the treated wastewater plastispheres showed more positive correlations between the taxa.

## Discussion

This study shows that genera that contain potential foodborne pathogenic taxa, such as *Salmonella* spp., *E*. *coli/Shigella*, *Listeria* spp., and *Campylobacter* spp., as well as potential opportunistic pathogens, such as genera *Klebsiella*, *Aeromonas*, *Serratia*, *Enterobacter*, and *Acinetobacter*, are found in plastispheres from raw and treated wastewater. These results are supported by previous studies showing that plastispheres from wastewater environments harbor diverse bacterial communities that include several genera associated with typical foodborne pathogens [22, 31, 34]. A biofilm can significantly increase the survival of many bacteria [62, 63]. Shen et al. (2021) showed via laboratory studies that MPs in a wastewater environment functioned as a protective habitat for pathogenic bacteria, increasing their survival in different WWT processes [64].

16S rRNA gene amplicon sequencing, used as a detection method in this study, is a high-throughput and sensitive method for microbial community analysis but does not provide information on the viability and pathogenicity of the bacteria. Therefore, complementary methods, such as cultivation, MALDI-TOF, and RT‒qPCR, were used.

The qPCR results confirmed the presence of enteropathogenic *E*. *coli* and *L*. *monocytogenes* but not *C*. *jejuni* in both the raw and treated wastewater plastispheres. These pathogens are among the most common causes of foodborne illnesses worldwide [65] and have previously been found in plastic-associated biofilms [31, 66, 67]. The absence of *C*. *jejuni* in the wastewater plastispheres was unexpected, as *Campylobacter* is commonly recovered from raw and treated wastewater [68, 69]. However, detection methods are often not sufficiently sensitive and appear to be challenging, and *C*. *jejuni* is often suppressed in the presence of competing organisms likely to be present in a biofilm [70, 71].

In contrast to the qPCR results, *E*. *coli*, *L*. *monocytogenes*, or any other species associated with foodborne bacterial pathogens were not isolated from the plastispheres by cultivation or MALDI-TOF. This suggests that they were either nonviable or present at levels below the detection limit of the method. Previous studies include an enrichment step to detect specific low-abundance pathogenic bacterial species or bacteria that require sensitive detection methods (such as *C*. *jejuni*) [72, 73]. We cannot exclude the possibility that viable species of *E*. *coli* or *L*. *monocytogenes* are present in the plastispheres and would have been detected by including enrichment in the analysis. On the other hand, other potential pathogenic bacteria, such as *K*. *pneumoniae* and *Acinetobacter* spp., were isolated and cultivated from both raw and treated wastewater plastispheres, indicating that these bacteria can survive on plastic surfaces. *K*. *pneumoniae* is ubiquitous and frequently found in treated wastewater. Although *K*. *pneumoniae* is not traditionally recognized as a foodborne pathogen, it has been recovered from food samples. Similarities between environmental and clinical isolates in WGS phylogenetic analysis [74, 75] suggest that some strains of *K*. *pneumoniae* could be classified as foodborne [76]. Kelly et al. (2021) reported higher levels of this bacterium on microplastics in wastewater effluent than in sewage [77]. Consequently, the presence of *K*. *pneumoniae* on microplastics in wastewater effluent raises concerns due to its clinical significance.

*Acinetobacter*, another opportunistic pathogen that causes hospital-acquired and nosocomial infections, has emerged as a multidrug-resistant threat worldwide [78]. The association of *Acinetobacter* with foodborne illness is scarce, some evidence suggests that fresh produce can be a vehicle for its transmission from the environment [79]. Carbapenem-resistant *Acinetobacter* has demonstrated significant persistence through various stages of wastewater treatment processes [80].

As mentioned, the most used method to detect pathogens in plastispheres is based on sequencing technologies [34, 77]. However, the usage of sequencing technologies to describe the risk associated with pathogens found in plastispheres has been criticized [81]. In our study, the detection of viable bacteria in the plastispheres emphasizes the importance of combining molecular techniques and culturing to identify pathogenic species in plastispheres. Previous studies have isolated viable *Klebsiella* spp. from plastispheres using enrichment and cultivation, which supports our results indicating that *K*. *pneumonia* can thrive in the plastispheres [72, 73]. Silva et al isolated *Acinetobacter* from freshwater plastispheres using cultivation techniques [82]. However, to our knowledge, there is a lack of studies on the survival of *Acinetobacter* spp. in plastispheres through the wastewater treatment process, underscoring the importance of our study.

Both treated and raw wastewater samples contained NoV (G1 and GII) and AdV (Table 2). The presence of norovirus in the plastispheres changed between D14 and D30, with an increase in plastispheres from raw wastewater. There was a lower level of NoV in plastispheres from treated wastewater than in those from raw wastewater. AdV and NoV are among the many viruses that can cause human infections via the fecal-oral route. NoVs are especially notorious for causing outbreaks of foodborne disease. According to data from the WHO involving 135 countries, NoVs account for most foodborne diseases [83]. In the US and UK, NoVs accounted for 58% (2000–2008) and 16% (2018) of foodborne diseases, respectively [84, 85]. NoV is also the main cause of acute gastroenteritis worldwide and is abundant in raw and treated wastewater [86]. AdV is mainly associated with gastroenteritis in children and is present at similar levels as NoV in wastewater [87]. These findings indicate that both NoV and AdV can adhere to and persist in the plastisphere. The implications of this phenomenon for the infectivity and transmission of these viruses are still unclear. A recent study showed that polystyrene induced the infectivity of the influenza A virus by affecting endocytosis and the innate antiviral immune system of human host cells, implicating the potential risk of viruses associated with plastic particles [88].

Although most microorganisms are effectively removed during the WWT process, plastic-associated biofilms in wastewater might provide a protective habitat for many pathogenic species, allowing them to survive the treatment process, as shown in this study [64, 77, 89, 90]. This highlights the importance of proper management and disposal of wastewater from WWTPs to prevent the environmental dissemination of these bacteria and viruses.

During wastewater treatment, large objects, and pollutants, including plastic particles, are removed to ensure that the effluent water discharged into the environment is free from biological and chemical contaminants [12, 91]. Most of this removal is performed in the primary treatment of raw wastewater. As a result, the number of plastic particles in raw wastewater exceeds that in treated wastewater. However, no treatment process is effective in completely removing the total load of both plastic particles and potential pathogenic microorganisms [77, 87, 89]. A 2014 report from the same WWTP as this study indicated that over 90% of plastic particles were eliminated during treatment [7]. However, more than 35 million plastic particles were still present in the discharged wastewater. Another recent report from a WWTP in Norway [11], estimated that 1.8 × 10^12^ plastic particles were present in the influent wastewater in one year, of which approximately 2.2 × 10^8^ plastic particles were released into the environment via the treated wastewater. Despite the effectiveness of WWTPs in removing plastic particles, a significant number of plastic particles are released into the environment. In addition, in the case of sewer overflow, plastic material harboring pathogenic microorganisms might be released directly into the riverine-coastal environment, bypassing several stages of the treatment within the WWTP, representing an indirect risk associated with plastispheres from wastewater [92, 93]. Continued research and innovation are necessary to develop alternative sustainable technologies or supplement conventional treatment processes to ensure more effective removal of microplastics and to degrade potential pathogenic microorganisms. Bioremediation and membrane bioreactors (MBR) are examples of promising methods for improved microplastic removal [94]. Wastewater surveillance detection through genetic sequences and biomarkers is suggested as an important tool for the early detection of pathogenic microorganisms [95, 96], although more research is needed to understand more about the risk of transmission of pathogens to humans through environmental exposure pathways [97].

Taken together, our genomic analysis and conventional cultivation results show the presence of potentially pathogenic bacteria and viruses in the plastispheres derived from wastewater. Several genera containing potentially pathogenic bacteria, in addition to NoV and AdV, were identified in the plastispheres formed from treated wastewater (Tables 1 and 2). Despite their low abundance, these results show that certain bacteria can survive wastewater treatment and thrive in plastispheres. This suggests that plastic surfaces can act as vectors for pathogenic microorganisms, and wastewater effluents containing these plastispheres could pose a risk for environmental contamination by these pathogens. There have been an increasing number of studies demonstrating that various microorganisms capable of causing disease in humans are present in the plastisphere [31, 64, 77, 89, 90]. The scientific understanding and evidence concerning plastic and microplastics acting as carriers for pathogens have changed accordingly. Several *in vitro* and *in vivo* studies have shown a higher absorption and increased mortality when organisms are exposed to pathogens associated with microplastics compared to sterile plastic particles [88, 98–100]. Altogether this establishes the presence of harmful pathogens in the plastispheres, which should not be ignored. The direct link between plastic-associated pathogens and human health remains unclear and not fully understood highlighting the need for further research. This adds to the concern related to the growing issue of increasing plastic pollution and the inadequate handling of plastic [3, 4] and calls for a reevaluation and potential update of regulatory frameworks to ensure the safety of wastewater reuse [101, 102].

### Bacterial community analysis

The results from the community composition and structure analyses (Fig 1, S4 and S5 Tables) as well as the diversity analysis (Tables 3 and 4, Figs 2 and 3) indicate differences in both composition and diversity between plastisphere communities from raw and treated wastewater.

In both raw and treated wastewater environments, plastisphere communities were dominated by the phyla Proteobacterium, followed by Bacteroidota and Firmicutes (Fig 1A). The co-occurrence network analysis also revealed that Proteobacterium had more interactions in both environments (Fig 4), suggesting an ecological importance and a significant role in the stability or function of the bacterial communities. These phyla are consistently found to be prevalent during all stages of wastewater treatment in previous studies [22, 103, 104]. Members of Proteobacteria are versatile and adaptable to various environmental conditions. They play crucial roles in nitrification and denitrification, contributing to the efficient removal of nitrogen from wastewater, and are commonly found at elevated levels in wastewater treatment facilities [104]. Furthermore, Proteobacteria, Firmicutes, and Bacteroidota include genera involved in the production of extracellular polymeric substances (EPS) that facilitate biofilm formation [105] and have plastic-degrading capabilities [77, 106]. The plastisphere communities of the raw wastewater were more diverse and contained more low-abundance taxa than the plastispheres from the treated wastewater (Fig 1B).

The plastisphere communities from treated wastewater were dominated by a few genera, such as *Methylotenera*, *Methanomethylovora*, and *Desulfosporosinus*. These compounds are normally found in WWTPs as part of the treatment process; are involved in biochemical cycles of carbon, nitrogen, and sulfur; and may have greater tolerance or resistance to the treatment process [107–110].

The diversity and richness of bacteria found in the plastispheres in this study are influenced by various factors, including the plastic material, the environmental conditions, and the time the plastic was submerged in the wastewater. The different types of plastic significantly affected the Shannon diversity but not the Chao1 richness. This indicates that different types of plastic may be selected for different bacterial communities in terms of composition but not necessarily in terms of abundance. PVC and HDPE had greater Shannon diversity in the plastispheres from the raw wastewater compared to PP, suggesting that these plastics may harbor more diverse bacterial taxa than PP. However, this significant difference was observed only in the plastispheres collected at D30 in the raw wastewater. These findings suggest that the type of plastic material affects the species diversity (Shannon) of the plastispheres, and this effect is dependent on the surrounding environment (raw or treated wastewater) and the duration of time the plastic pieces were submerged in these specific environments. This contradicts the results from our previous study indicating that the type of plastic did not have an impact on the bacterial diversity in plastispheres from river water [31].

The photochemical and biological degradation of plastics may lead to the leaching of dissolved organic substances [111], which provide nutrients that promote bacterial growth [111, 112]. On the other hand, certain hazardous chemicals added to synthetic polymers during production, such as those that improve plastic flexibility and heat stability, may inhibit bacterial growth in plastic leachate [113]. Additionally, the initial attachment of microorganisms and the formation of biofilms are influenced by the physicochemical characteristics of plastic surfaces, such as roughness, hydrophobicity, topography, and electrostatic interactions [27, 114, 115]. However, other reports indicate no significant effect of the surface topography or roughness of the polymer on the initial attachment of microorganisms [116, 117] or the bacterial community structure and composition [31, 118, 119]. Plastic found in wastewater comprises several plastic materials [11, 14]. This study specifically analyzed the bacterial communities on three different plastic materials, chosen to reflect the plastic usually found in plastic pollution in different environments and represent materials usually found in WWTP [40, 41]. Each material selected for the study exhibits unique characteristics and chemical compositions, providing insight into how these differences might influence biofilm formation. However, the results from this study are limited to these substrates. Further research including additional plastic types would be necessary to generalize the findings more broadly across all plastics encountered in wastewater.

The environment had a significant effect on the alpha diversity indices analyzed, with the Shannon diversity and Chao1 richness of the raw wastewater plastispheres being greater than those of the treated wastewater plastispheres. Moreover, a change in the composition of the bacterial community in the plastispheres was observed. This shift is also evident in the PCoA plot, where the bacterial communities in the plastispheres clustered according to the two environments (Fig 3). One potential explanation for this phenomenon lies in the higher level of organic matter and nutrient content present in the raw wastewater, providing more resources for bacterial growth and colonization. Consequently, this environment might favor certain bacterial taxa and promote biofilm formation. The formation of biofilms is highly affected by the environment, and the decision of bacteria to either form or disperse from a biofilm is determined by environmental factors [120]. Raw wastewater consists of household sewage, industrial or hospital wastewater, and urban runoff [12] and consequently contains a diverse microbiome originating from many different sources [20, 21].

The co-occurrence network analysis revealed more negative correlations in the raw wastewater plastispheres (Fig 4). This may be a result of the environmental conditions in the raw wastewater. The diversity analysis showed that the raw wastewater plastispheres exhibited higher diversity and richness. These findings suggest that the heterogeneous and variable conditions in raw wastewater promote increased competition among microbial species. The competitive interaction could also reflect a more resilient community due to high diversity. Each taxon has its unique role, contributing to the overall function of the ecosystem’s function. On the other hand, the diversity and richness in the treated wastewater plastispheres were lower, and the network analysis revealed more positive correlations than the raw wastewater plastispheres. This might indicate a more cooperative or mutualistic interaction in the treated wastewater plastispheres, reflecting a more uniform environment in the treated wastewater.

One purpose of the wastewater treatment process is to remove microorganisms. Thus, compared with treated wastewater, raw wastewater is expected to contain a broader spectrum of bacterial taxa. The difference observed between the plastispheres from the raw and treated wastewater in this study may be due to variations in the diversity and composition of the wastewater microbiome [121, 122].

Our results show that the surrounding environment seems to have a great impact on the composition of the plastispheres, which is also shown in other studies on plastispheres from marine, fresh, and wastewater environments [123–125]. In addition, there is also a discussion regarding a plastisphere “core microbiome”, which is also dependent on the environment [23, 24]. Several studies have compared the plastispheres with the surrounding water environments and other surfaces [25, 123, 126], suggesting that plastic surface contributes to a unique bacterial niche that is prone to be colonized by bacteria other than those contributing to planktonic communities [35, 127, 128]. Results indicate that the plastisphere contains a unique taxonomy compared to the planktonic microbiome or other biotic surfaces in the surrounding environment. We recognize this as valuable information for understanding the complexity and the impact of the plastispheres. One of the aims of this study was to describe the bacterial community composition of the plastispheres from raw and treated wastewater. To perform a comparative analysis of the bacterial composition on plastic compared to other bacterial communities was beyond the scope of our study. Also, it is important to note that the wastewater basins are dynamic environments with a continuous wastewater flow. Our sampling period lasted up to four weeks. Thus, the “snapshot” water samples will not fully capture the complexity and dynamics of the surrounding environment when compared to the plastisphere microbiome. This is an essential aspect to consider when interpreting the results and understanding the correlation between the plastispheres and their microbial composition in the wastewater system.

The duration of incubation had a significant effect on the Shannon diversity index but not on the Chao1 richness index. The Shannon diversity increased from D14 to D30 in treated wastewater, showing that the bacterial communities became more diverse over time in this environment. This is evident in Fig 3, which illustrates a clear clustering of the bacterial communities according to the day of sampling. Changes in the composition of the plastisphere communities over time were also evident in our previous study showing an increase in Shannon diversity between plastispheres collected after two and four weeks of incubation in river water [31]. This phenomenon may be attributed to the replacement of certain bacterial taxa with others within the environment. Biofilms are dynamic structures shaped by various physical and chemical parameters in the surrounding environment [119, 129]. Zhang et al. observed a primary succession pattern in biofilm communities in wastewater distribution systems, with the taxonomic composition changing over time [130].

This study shed light on the plastispheres in wastewater environments. The findings focus on the variability in plastispheres from raw and treated wastewater. The environments had a significant impact on the diversity of the bacteria in the plastispheres, emphasizing the significant role of the treatment process in shaping the microbial composition of these plastic-associated communities. Potentially foodborne pathogenic microorganisms such as *L*. *monocytogenes*, *E*. *coli*, and adeno- and norovirus were detected in the plastispheres from wastewater. Crucially, viable isolates of the emerging pathogens *K*. *pneumonia* and *Acinetobacter* were detected in the plastispheres from treated wastewater. Altogether, our findings underscore the role of plastispheres originating from wastewater as reservoirs for bacteria and viruses, addressing one problematic aspect of the lack of proper handling of plastic waste. This highlights the potential for plastic particles to disperse pathogens from highly polluted environments as wastewater to other aquatic ecosystems or even transfer them to other ecological niches. Our research contributes to the knowledge base in this field. However, more research and innovation, in addition to effective measures or improved wastewater management, are needed to prevent or minimize the release of plastic pollutants from WWTP into aquatic ecosystems.

## Supporting information

S1 Fig Biofilm formation on plastic pieces submerged in wastewater. Representative pictures of biofilm on plastic pieces from wastewater. Top = HDPE, middle = PP, bottom = PVC. These pieces have been submerged in the treated wastewater for 14 days.
(TIF)

S1 Table The selective media and growth conditions used to cultivate the potential pathogenic bacteria. (DOCX)

S2 Table Sample information. Several variables were considered for the bacterial diversity analysis; plastic type, duration of incubation (14 days (D14) or 30 days (D30)), and environment (raw or treated wastewater) The samples were organized into groups according to combinations of these variables, with three replicates within each group.
(DOCX)

S3 Table The primers and probes used for molecular detection of viruses and bacteria. (DOCX)

S4 Table The most abundant phyla in the wastewater plastispheres. The 20 most abundant phyla in the plastispheres from raw and treated wastewater. The mean and standard deviations are calculated based on the abundance of each phylum across the variables.
(DOCX)

S5 Table The most abundant genera in the wastewater plastispheres. The abundance of the 20 most abundant genera in plastispheres from raw and treated wastewater. The mean and standard deviations are calculated based on the abundance of each genus across the variables.
(DOCX)
