# Supplementary figures and images for: Wastewater-associated plastispheres: A hidden habitat for microbial pathogens?
Source: PLoS One. 2024 Nov 6;19(11):e0312157. doi: 10.1371/journal.pone.0312157 (PMC11540174; doi:10.1371/journal.pone.0312157)

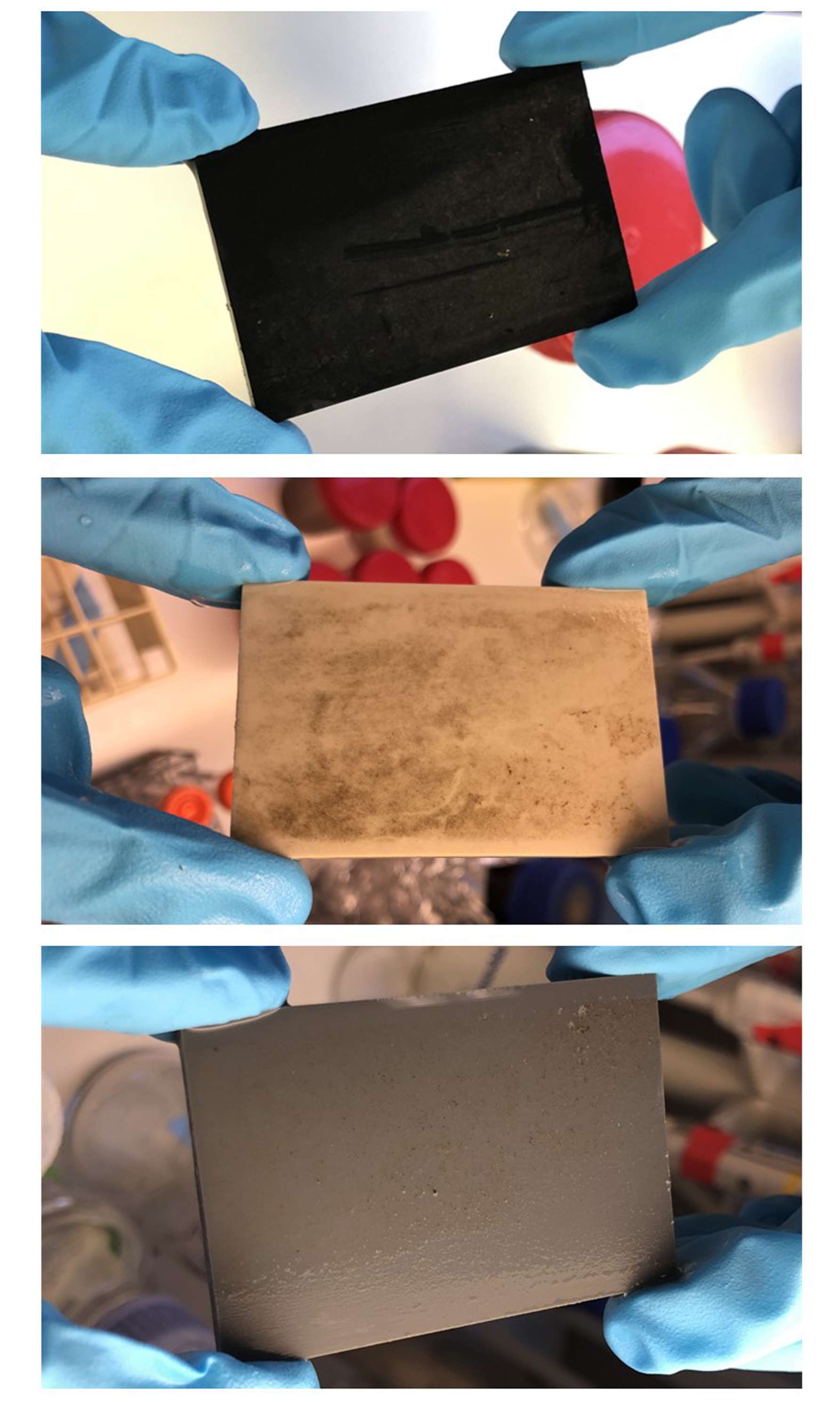

Supplement: S1 Fig — Representative pictures of biofilm on plastic pieces from wastewater. Top = HDPE, middle = PP, bottom = PVC. These pieces have been submerged in the treated wastewater for 14 days. (TIF) [file pone.0312157.s001.tif]
