# Supplementary material for: Wastewater-associated plastispheres: A hidden habitat for microbial pathogens?
Source: PLoS One. 2024 Nov 6;19(11):e0312157. doi: 10.1371/journal.pone.0312157 (PMC11540174; doi:10.1371/journal.pone.0312157)
Supplement: S1 Table — (DOCX) [file pone.0312157.s002.docx]

**S1 Table. The selective media and growth conditions used to cultivate the potential pathogenic bacteria.**

| Species |  | Selective media | Incubation condition | Colony morphology |
| --- | --- | --- | --- | --- |
| *Aeromonas* sp | Enrichment | Alkaline Peptone water | 35°C, 8 h,  aerobically |  |
|  | Selective plating | Aeromonas Yersinia agar | 35°C, 48 h, aerobically | colourless and pale red, with rose to red center |
| *Campylobacter* sp | Enrichment 1 | Bolton broth with selective supplement SR0183 | 37°C, 4 h, microaerobic^1)^ |  |
|  | Enrichment 2 | Bolton broth with selective supplement SR0183^2)^ | 42°C, 48 h, microaerobic^1)^ |  |
|  | Selective plating | Charcoal Cefoperazone Deoxychoclate (CCDA) | 42°C, 48 h, microaerobic^1)^ | grey, white, creamy with moist appearance |
| *Escherichia coli* O157) | Enrichment | Tryptone Soyabroth with novobiocin (29 mg/L) | 41.5°C, 6 h, aerobically |  |
|  | Selective plating | Sorbitol MacConkey Agar with Cefixime potassium tellurite (SMAC-CT) | 37°C, 24 h, aerobically | colourless colonies |
| *Listeria monocytogenes* | Enrichment1 | Half Fraser broth | 30°C, 24 h, aerobically |  |
|  | Enrichment2 | Fraser both | 37°C, 24 h, aerobically |  |
|  | Selective plating | ALOA plates | 37°C, 24 h, aerobically | green-blue colonies surrounded by opaque halo |
| *Salmonella* | Enrichment 1 | Buffered Peptone Water | 37°C, 24 h, aerobically |  |
|  | Enrichment 2 | Muller-Kauffmann Tetrathionate Novobiocin broth (MKTTn) | 37°C, 24 h, aerobically |  |
|  | Selective plating | Xylose Lysine Desoxycholate Agar (XLD) | 37°C, 24 h, aerobically | pink/red with black center |
