# Supplementary material for: Wastewater-associated plastispheres: A hidden habitat for microbial pathogens?
Source: PLoS One. 2024 Nov 6;19(11):e0312157. doi: 10.1371/journal.pone.0312157 (PMC11540174; doi:10.1371/journal.pone.0312157)
Supplement: S2 Table — Several variables were considered for the bacterial diversity analysis; plastic type, duration of incubation (14 days (D14) or 30 days (D30)), and environment (raw or treated wastewater) The samples were organized into groups according to combinations of these variables, with three replicates within each group. (DOCX) [file pone.0312157.s003.docx]

**S2 Table.** **Sample information.** Several variables were considered for the bacterial diversity analysis; plastic-type, duration of incubation (14 days (D14) or 30 days (D30)), and environment (raw or treated wastewater) The samples were organized into groups according to combinations of these variables, with three replicates within each group.

|  |  | **D14** | | **D30** | |
| --- | --- | --- | --- | --- | --- |
| **Environment** | **Plastic type** | **Sample ID** | **Group ID** | **Sample ID** | **Group ID** |
| **Raw wastewater** | **PP** | F54 | A2 | F13 | A4 |
|  |  | F55 |  | F14 |  |
|  |  | F56 |  | F15 |  |
|  | **PVC** | F57 | B2 | F16 | B4 |
|  |  | F58 |  | F17 |  |
|  |  | F59 |  | F18 |  |
|  | **HDPE** | F60 | C2 | F20 | C4 |
|  |  | F61 |  | F21 |  |
|  |  | F62 |  | F22 |  |
| **Treated wastewater** | **PP** | E44 | D2 | E34 | D4 |
|  |  | E45 |  | E35 |  |
|  |  | E46 |  | E36 |  |
|  | **PVC** | E47 | E2 | E37 | E4 |
|  |  | E48 |  | E38 |  |
|  |  | E49 |  | E39 |  |
|  | **HDPE** | E50 | F2 | E40 | F4 |
|  |  | E51 |  | E42 |  |
|  |  | E52 |  | E43 |  |
