# Supplementary material for: Wastewater-associated plastispheres: A hidden habitat for microbial pathogens?
Source: PLoS One. 2024 Nov 6;19(11):e0312157. doi: 10.1371/journal.pone.0312157 (PMC11540174; doi:10.1371/journal.pone.0312157)
Supplement: S3 Table — (DOCX) [file pone.0312157.s004.docx]

**S3 Table.** **Primers and probes used for molecular detection of viruses and bacteria.**

| **Primers and probes** | **Sequences (5’-3’)** | **References** |
| --- | --- | --- |
| Adenovirus  JTVXF  JTVXR  JTVXP | GGACGCCTCGGAGTACCTGAG ACIGTGGGGTTTCTGAACTTGTT CTGGTGCAGTTCGCCCGTGCCA | (Jothikumar et al. 2005) |
| Norovirus GI  QNIF4fw  NV1LCRrv  NVGG1p | CGCTGGATGCGNTTCCAT  CCTTAGACGCCATCATCATTTAC  TGGACAGGAGAYCGCRATCT | (Silva et al. 2007; Svraka et al. 2007) |
| Norovirus GII  QNIF2 fw  COG2Rrv  QNIFsp | ATGTTCAGRTGGATGAGRTTCTCWGA  TCGACGCCATCTTCATTCACA  AGCACGTGGGAGGGCGATCG | (Kageyama et al. 2003; Loisy et al. 2005) |
| EPEC  *Tir* | GTCAGCTCATTAACTCTACGGG  GCCTGTTAAGAGTATCGAGCG | (Haffar & Gilbride 2010) |
| *L. monocytogenes*  *hlyA* | GGGAAATCTGTCTCAGGTGATGT  CGATGATTTGAACTTCATCTTTTGC | (Guilbaud et al. 2005) |
| *C. jejuni*  VS1 | GAATGAAATTTTAGAATGGGG  CGATGATTTGAACTTCATCTTTTGC | (Stonnet, V. & Guesdon, J.-L. 1993) |
