# Supplementary material for: Wastewater-associated plastispheres: A hidden habitat for microbial pathogens?
Source: PLoS One. 2024 Nov 6;19(11):e0312157. doi: 10.1371/journal.pone.0312157 (PMC11540174; doi:10.1371/journal.pone.0312157)
Supplement: S4 Table — The 20 most abundant phyla in the plastispheres from raw and treated wastewater. The mean and standard deviations are calculated based on the abundance of each phylum across the variables. (DOCX) [file pone.0312157.s005.docx]

**S4 Table. The most abundant phyla in the wastewater plastispheres.**

The 20 most abundant phyla in the plastispheres from raw and treated wastewater. The mean and standard deviations are calculated based on the abundance of each phylum across the variables.

|  | **Raw wastewater** | | | | | | | | |  |  |
| --- | --- | --- | --- | --- | --- | --- | --- | --- | --- | --- | --- |
| **D14** | **PP** | **PP** | **PP** | **PVC** | **PVC** | **PVC** | **HDPE** | **HDPE** | **HDPE** | **Mean** | **St.dev** |
| Proteobacteria | 29.088% | 45.964% | 42.178% | 45.929% | 39.499% | 42.816% | 40.816% | 38.170% | 42.038% | 40.722% | 4.787% |
| Firmicutes | 34.104% | 21.252% | 19.436% | 23.586% | 23.677% | 25.134% | 23.115% | 30.825% | 25.562% | 25.188% | 4.335% |
| Halobacterota | 0.005% | 0.090% | 0.112% | 0.096% | 0.088% | 0.071% | 0.066% | 0.033% | 0.066% | 0.070% | 0.031% |
| Desulfobacterota | 9.690% | 10.123% | 8.784% | 5.756% | 6.548% | 5.619% | 8.890% | 6.118% | 6.362% | 7.543% | 1.698% |
| Bacteroidota | 14.570% | 9.227% | 10.115% | 10.589% | 14.126% | 11.570% | 9.847% | 15.510% | 11.079% | 11.848% | 2.164% |
| Actinobacteriota | 6.523% | 4.312% | 5.542% | 6.337% | 6.268% | 6.816% | 4.668% | 3.825% | 5.937% | 5.581% | 1.006% |
| Patescibacteria | 0.326% | 0.186% | 0.233% | 0.342% | 0.389% | 0.258% | 0.211% | 0.255% | 0.299% | 0.278% | 0.062% |
| Campilobacterota | 2.299% | 1.844% | 2.416% | 2.197% | 1.912% | 1.953% | 5.027% | 3.312% | 6.321% | 3.031% | 1.502% |
| Acidobacteriota | 0.277% | 1.441% | 2.997% | 0.795% | 1.874% | 1.310% | 1.805% | 0.151% | 0.153% | 1.200% | 0.903% |
| Verrucomicrobiota | 0.052% | 0.068% | 0.301% | 0.068% | 0.181% | 0.110% | 0.162% | 0.077% | 0.019% | 0.115% | 0.082% |
| Chloroflexi | 0.101% | 0.879% | 1.740% | 0.655% | 1.214% | 0.696% | 0.986% | 0.090% | 0.082% | 0.716% | 0.534% |
| Nitrospirota | 0.121% | 0.811% | 0.973% | 0.523% | 0.682% | 0.542% | 0.608% | 0.121% | 0.173% | 0.506% | 0.291% |
| Fusobacteriota | 0.534% | 0.101% | 0.151% | 1.066% | 0.249% | 0.608% | 0.156% | 0.485% | 0.493% | 0.427% | 0.288% |
| Myxococcota | 0.164% | 0.992% | 1.044% | 0.304% | 0.562% | 0.471% | 0.504% | 0.077% | 0.085% | 0.467% | 0.339% |
| Spirochaetota | 0.249% | 0.208% | 0.241% | 0.099% | 0.142% | 0.140% | 0.211% | 0.112% | 0.071% | 0.164% | 0.061% |
| Synergistota | 0.367% | 0.438% | 0.359% | 0.452% | 0.444% | 0.337% | 0.299% | 0.370% | 0.348% | 0.379% | 0.050% |
| Sva0485 | 0.011% | 0.318% | 0.455% | 0.208% | 0.334% | 0.290% | 0.356% | 0.000% | 0.000% | 0.219% | 0.164% |
| Nitrospinota | 0.003% | 0.301% | 0.534% | 0.115% | 0.351% | 0.200% | 0.342% | 0.005% | 0.000% | 0.206% | 0.179% |
| Methylomirabilota | 0.008% | 0.216% | 0.364% | 0.181% | 0.132% | 0.096% | 0.197% | 0.000% | 0.005% | 0.133% | 0.114% |
| Gemmatimonadota | 0.049% | 0.126% | 0.263% | 0.079% | 0.227% | 0.142% | 0.214% | 0.033% | 0.038% | 0.130% | 0.082% |
| **D30** | **PP** | **PP** | **PP** | **PVC** | **PVC** | **PVC** | **HDPE** | **HDPE** | **HDPE** | **Mean** | **St.dev** |
| Proteobacteria | 56.350% | 50.337% | 23.214% | 41.290% | 43.805% | 50.334% | 42.852% | 45.479% | 36.699% | 43.373% | 8.979% |
| Firmicutes | 19.611% | 22.649% | 26.773% | 20.236% | 17.655% | 20.518% | 20.879% | 22.208% | 22.321% | 21.428% | 2.393% |
| Halobacterota | 0.219% | 0.164% | 0.211% | 1.233% | 1.036% | 0.134% | 0.611% | 0.195% | 1.504% | 0.590% | 0.503% |
| Desulfobacterota | 3.567% | 6.411% | 19.162% | 15.595% | 8.526% | 6.173% | 7.384% | 7.356% | 12.764% | 9.660% | 4.793% |
| Bacteroidota | 7.775% | 8.011% | 16.126% | 8.227% | 8.775% | 8.764% | 9.756% | 8.816% | 8.953% | 9.467% | 2.418% |
| Actinobacteriota | 6.945% | 5.819% | 6.748% | 3.789% | 6.518% | 7.551% | 8.175% | 7.847% | 4.170% | 6.396% | 1.458% |
| Patescibacteria | 0.225% | 0.438% | 0.923% | 0.584% | 0.540% | 0.252% | 0.605% | 0.416% | 0.732% | 0.524% | 0.210% |
| Campilobacterota | 2.973% | 3.795% | 3.389% | 1.268% | 1.784% | 1.227% | 1.521% | 1.652% | 2.603% | 2.246% | 0.910% |
| Acidobacteriota | 0.170% | 0.167% | 0.173% | 1.340% | 2.940% | 0.921% | 2.060% | 1.479% | 2.263% | 1.279% | 0.954% |
| Verrucomicrobiota | 0.090% | 0.063% | 0.134% | 0.123% | 0.364% | 0.074% | 0.233% | 0.096% | 0.334% | 0.168% | 0.108% |
| Chloroflexi | 0.255% | 0.247% | 0.211% | 0.984% | 1.567% | 0.937% | 1.389% | 0.882% | 1.216% | 0.854% | 0.482% |
| Nitrospirota | 0.345% | 0.384% | 0.134% | 0.685% | 1.186% | 0.438% | 0.923% | 0.570% | 0.838% | 0.612% | 0.310% |
| Fusobacteriota | 0.099% | 0.268% | 0.290% | 0.044% | 0.167% | 0.808% | 0.326% | 0.477% | 0.090% | 0.286% | 0.225% |
| Myxococcota | 0.101% | 0.129% | 0.178% | 0.964% | 0.923% | 0.301% | 0.553% | 0.381% | 0.770% | 0.478% | 0.320% |
| Spirochaetota | 0.110% | 0.263% | 0.926% | 0.468% | 0.321% | 0.153% | 0.222% | 0.184% | 0.515% | 0.351% | 0.241% |
| Synergistota | 0.175% | 0.225% | 0.356% | 0.614% | 0.342% | 0.186% | 0.271% | 0.241% | 0.679% | 0.343% | 0.173% |
| Sva0485 | 0.005% | 0.003% | 0.008% | 0.296% | 0.564% | 0.266% | 0.378% | 0.299% | 0.468% | 0.254% | 0.196% |
| Nitrospinota | 0.016% | 0.000% | 0.000% | 0.301% | 0.474% | 0.115% | 0.285% | 0.156% | 0.384% | 0.192% | 0.166% |
| Methylomirabilota | 0.000% | 0.005% | 0.005% | 0.173% | 0.307% | 0.186% | 0.164% | 0.115% | 0.173% | 0.125% | 0.098% |
| Gemmatimonadota | 0.066% | 0.030% | 0.019% | 0.096% | 0.279% | 0.058% | 0.211% | 0.107% | 0.318% | 0.132% | 0.104% |

|  | **Treated wastewater** | | | | | | | | | | |
| --- | --- | --- | --- | --- | --- | --- | --- | --- | --- | --- | --- |
| **D14** | **PP** | **PP** | **PP** | **PVC** | **PVC** | **PVC** | **HDPE** | **HDPE** | **HDPE** | **Mean** | **St.dev** |
| Proteobacteria | 74.241% | 74.359% | 75.858% | 74.581% | 73.082% | 73.682% | 72.836% | 74.416% | 70.827% | 73.765% | 1.334% |
| Firmicutes | 5.195% | 4.562% | 3.227% | 4.184% | 4.310% | 4.178% | 4.227% | 3.342% | 5.907% | 4.348% | 0.784% |
| Halobacterota | 1.342% | 0.866% | 0.553% | 0.649% | 0.904% | 0.967% | 1.214% | 0.734% | 1.299% | 0.948% | 0.268% |
| Desulfobacterota | 0.937% | 0.751% | 0.630% | 0.836% | 0.926% | 1.079% | 0.964% | 0.764% | 0.970% | 0.873% | 0.131% |
| Bacteroidota | 10.397% | 9.211% | 8.830% | 10.748% | 10.348% | 9.515% | 9.868% | 9.099% | 12.452% | 10.052% | 1.048% |
| Actinobacteriota | 1.548% | 1.573% | 1.288% | 1.441% | 1.652% | 1.556% | 1.282% | 1.274% | 1.841% | 1.495% | 0.182% |
| Patescibacteria | 2.304% | 4.386% | 5.926% | 3.447% | 4.145% | 3.460% | 5.252% | 6.438% | 2.529% | 4.210% | 1.360% |
| Campilobacterota | 0.553% | 0.704% | 0.871% | 0.896% | 1.132% | 1.271% | 0.940% | 0.849% | 0.674% | 0.877% | 0.211% |
| Acidobacteriota | 0.277% | 0.252% | 0.162% | 0.200% | 0.181% | 0.252% | 0.225% | 0.216% | 0.208% | 0.219% | 0.035% |
| Verrucomicrobiota | 0.510% | 0.704% | 0.203% | 0.403% | 0.310% | 0.367% | 0.353% | 0.277% | 0.367% | 0.388% | 0.137% |
| Chloroflexi | 0.268% | 0.299% | 0.170% | 0.247% | 0.225% | 0.279% | 0.236% | 0.301% | 0.395% | 0.269% | 0.059% |
| Cyanobacteria | 0.184% | 0.356% | 0.447% | 0.392% | 0.477% | 0.625% | 0.419% | 0.504% | 0.142% | 0.394% | 0.143% |
| Nitrospirota | 0.929% | 0.732% | 0.663% | 0.789% | 0.921% | 1.153% | 0.778% | 0.696% | 1.011% | 0.852% | 0.153% |
| Myxococcota | 0.745% | 0.701% | 0.630% | 0.685% | 0.682% | 0.789% | 0.663% | 0.548% | 0.726% | 0.686% | 0.066% |
| Spirochaetota | 0.096% | 0.068% | 0.049% | 0.085% | 0.052% | 0.090% | 0.082% | 0.099% | 0.126% | 0.083% | 0.023% |
| Cloacimonadota | 0.005% | 0.008% | 0.016% | 0.011% | 0.003% | 0.022% | 0.019% | 0.022% | 0.041% | 0.016% | 0.011% |
| LCP-89 | 0.005% | 0.000% | 0.019% | 0.019% | 0.003% | 0.025% | 0.016% | 0.011% | 0.019% | 0.013% | 0.008% |
| Gemmatimonadota | 0.101% | 0.082% | 0.036% | 0.068% | 0.077% | 0.063% | 0.085% | 0.063% | 0.110% | 0.076% | 0.021% |
| Bdellovibrionota | 0.107% | 0.093% | 0.115% | 0.088% | 0.123% | 0.137% | 0.156% | 0.115% | 0.082% | 0.113% | 0.023% |
| Deferribacterota | 0.000% | 0.077% | 0.088% | 0.008% | 0.184% | 0.189% | 0.134% | 0.016% | 0.005% | 0.078% | 0.072% |
| **D30** | **PP** | **PP** | **PP** | **PVC** | **PVC** | **PVC** | **HDPE** | **HDPE** | **HDPE** | **Mean** | **St.dev** |
| Proteobacteria | 57.660% | 55.118% | 50.784% | 49.899% | 51.227% | 55.534% | 61.101% | 52.660% | 45.216% | 53.244% | 4.423% |
| Firmicutes | 9.753% | 10.016% | 11.175% | 10.197% | 10.995% | 10.499% | 8.652% | 9.101% | 8.129% | 9.835% | 0.978% |
| Halobacterota | 9.222% | 9.786% | 14.334% | 12.085% | 13.633% | 5.926% | 7.367% | 17.844% | 26.537% | 12.970% | 5.926% |
| Desulfobacterota | 1.863% | 2.227% | 1.951% | 3.071% | 2.455% | 2.625% | 2.066% | 1.912% | 1.973% | 2.238% | 0.383% |
| Bacteroidota | 10.995% | 11.899% | 11.359% | 12.923% | 11.463% | 12.795% | 12.764% | 10.085% | 10.564% | 11.650% | 0.967% |
| Actinobacteriota | 0.868% | 0.901% | 0.482% | 1.123% | 0.921% | 1.112% | 0.833% | 1.260% | 0.770% | 0.919% | 0.216% |
| Patescibacteria | 2.742% | 2.400% | 2.044% | 2.362% | 2.334% | 3.216% | 1.890% | 2.386% | 2.381% | 2.417% | 0.361% |
| Campilobacterota | 2.038% | 2.923% | 2.507% | 1.992% | 1.723% | 2.403% | 1.173% | 0.789% | 0.882% | 1.826% | 0.705% |
| Acidobacteriota | 0.225% | 0.219% | 0.126% | 0.359% | 0.255% | 0.255% | 0.304% | 0.321% | 0.238% | 0.256% | 0.064% |
| Verrucomicrobiota | 0.427% | 0.570% | 2.427% | 0.633% | 0.548% | 0.485% | 0.479% | 0.397% | 0.595% | 0.729% | 0.605% |
| Chloroflexi | 0.288% | 0.203% | 0.205% | 0.353% | 0.285% | 0.362% | 0.337% | 0.296% | 0.268% | 0.289% | 0.054% |
| Cyanobacteria | 1.216% | 0.953% | 0.222% | 1.337% | 1.038% | 1.145% | 0.397% | 0.753% | 0.151% | 0.802% | 0.419% |
| Nitrospirota | 0.386% | 0.414% | 0.271% | 0.553% | 0.384% | 0.342% | 0.630% | 0.501% | 0.603% | 0.454% | 0.117% |
| Myxococcota | 0.474% | 0.532% | 0.427% | 0.532% | 0.559% | 0.589% | 0.559% | 0.521% | 0.405% | 0.511% | 0.059% |
| Spirochaetota | 0.290% | 0.353% | 0.285% | 0.455% | 0.342% | 0.523% | 0.247% | 0.230% | 0.288% | 0.335% | 0.092% |
| Cloacimonadota | 0.477% | 0.288% | 0.493% | 0.419% | 0.474% | 0.849% | 0.260% | 0.222% | 0.288% | 0.419% | 0.181% |
| LCP-89 | 0.200% | 0.285% | 0.203% | 0.304% | 0.340% | 0.433% | 0.148% | 0.066% | 0.107% | 0.232% | 0.112% |
| Gemmatimonadota | 0.096% | 0.052% | 0.016% | 0.058% | 0.068% | 0.044% | 0.055% | 0.085% | 0.044% | 0.058% | 0.022% |
| Bdellovibrionota | 0.195% | 0.219% | 0.148% | 0.260% | 0.205% | 0.274% | 0.140% | 0.167% | 0.145% | 0.195% | 0.047% |
| Deferribacterota | 0.096% | 0.003% | 0.000% | 0.159% | 0.112% | 0.036% | 0.003% | 0.033% | 0.008% | 0.050% | 0.055% |
