# Supplementary material for: Wastewater-associated plastispheres: A hidden habitat for microbial pathogens?
Source: PLoS One. 2024 Nov 6;19(11):e0312157. doi: 10.1371/journal.pone.0312157 (PMC11540174; doi:10.1371/journal.pone.0312157)
Supplement: S5 Table — The abundance of the 20 most abundant genera in plastispheres from raw and treated wastewater. The mean and standard deviations are calculated based on the abundance of each genus across the variables. (DOCX) [file pone.0312157.s006.docx]

**S5 Table. The most abundant genera in the wastewater plastispheres.**

The abundance of the 20 most abundant genera in plastispheres from raw and treated wastewater. The mean and standard deviations are calculated based on the abundance of each genus across the variables.

|  | **Raw wastewater** | | | | | | | | |  |  |
| --- | --- | --- | --- | --- | --- | --- | --- | --- | --- | --- | --- |
| **2 weeks** | **PP** | **PP** | **PP** | **PVC** | **PVC** | **PVC** | **HDPE** | **HDPE** | **HDPE** | **Mean** | **St.dev** |
| *Methylotenera* | 0.964% | 1.238% | 1.822% | 1.288% | 1.288% | 1.268% | 1.164% | 1.310% | 1.633% | 1.331% | 0.238% |
| *Methanomethylovorans* | 0.003% | 0.011% | 0.025% | 0.014% | 0.022% | 0.011% | 0.011% | 0.008% | 0.016% | 0.013% | 0.006% |
| *Acinetobacter* | 5.233% | 7.742% | 8.844% | 8.518% | 6.962% | 6.126% | 10.027% | 4.671% | 10.463% | 7.621% | 1.922% |
| *Zoogloea* | 3.233% | 2.570% | 3.041% | 2.975% | 2.545% | 2.474% | 6.307% | 4.444% | 5.307% | 3.655% | 1.300% |
| *Desulfobulbus* | 5.153% | 1.622% | 1.726% | 1.101% | 1.395% | 1.307% | 2.101% | 1.718% | 1.419% | 1.949% | 1.165% |
| *Comamonas* | 0.436% | 9.433% | 0.726% | 4.066% | 1.041% | 1.036% | 0.721% | 0.556% | 0.740% | 2.084% | 2.802% |
| *Hyphomicrobium* | 0.468% | 0.597% | 0.649% | 0.868% | 0.575% | 0.638% | 0.395% | 0.537% | 0.504% | 0.581% | 0.127% |
| *Desulfosporosinus* | 0.000% | 0.000% | 0.005% | 0.000% | 0.000% | 0.008% | 0.003% | 0.000% | 0.005% | 0.002% | 0.003% |
| *Bacillus* | 4.584% | 2.775% | 2.967% | 2.605% | 2.984% | 3.170% | 2.868% | 6.995% | 2.641% | 3.510% | 1.354% |
| *Neisseria* | 1.748% | 0.805% | 0.063% | 2.633% | 1.323% | 6.005% | 0.077% | 2.885% | 1.318% | 1.873% | 1.730% |
| *Gracilibacteria* | 0.019% | 0.016% | 0.014% | 0.044% | 0.033% | 0.019% | 0.019% | 0.005% | 0.038% | 0.023% | 0.012% |
| *Rhodoferax* | 0.312% | 0.405% | 0.438% | 0.499% | 0.405% | 0.474% | 0.348% | 0.370% | 0.444% | 0.411% | 0.057% |
| *Streptococcus* | 4.685% | 1.236% | 1.184% | 2.332% | 3.345% | 4.200% | 4.764% | 2.737% | 5.507% | 3.332% | 1.481% |
| *Macellibacteroides* | 4.510% | 2.682% | 2.978% | 3.268% | 3.912% | 3.400% | 3.041% | 5.353% | 3.863% | 3.668% | 0.797% |
| *Christensenellaceae_R-7_group* | 2.649% | 1.099% | 1.271% | 1.047% | 1.112% | 0.956% | 0.989% | 0.737% | 0.781% | 1.182% | 0.541% |
| *Acidovorax* | 1.622% | 2.033% | 2.151% | 2.474% | 2.101% | 2.436% | 2.033% | 1.973% | 2.638% | 2.162% | 0.291% |
| *Thiothrix* | 0.036% | 0.027% | 0.038% | 0.041% | 0.022% | 0.033% | 0.019% | 0.030% | 0.022% | 0.030% | 0.007% |
| *Sphaerotilus* | 0.260% | 0.025% | 0.055% | 0.047% | 0.036% | 0.071% | 0.033% | 0.027% | 0.036% | 0.065% | 0.070% |
| *Salmonella* | 0.247% | 0.208% | 0.230% | 0.214% | 0.197% | 0.211% | 0.211% | 4.170% | 0.211% | 0.655% | 1.243% |
| *Hydrogenophaga* | 0.233% | 0.216% | 0.241% | 0.225% | 0.225% | 0.427% | 0.378% | 0.310% | 0.337% | 0.288% | 0.074% |
| **4 weeks** | **PP** | **PP** | **PP** | **PVC** | **PVC** | **PVC** | **HDPE** | **HDPE** | **HDPE** | **Mean** | **St.dev** |
| *Methylotenera* | 1.121% | 1.833% | 0.751% | 0.838% | 1.685% | 1.033% | 1.532% | 1.460% | 0.948% | 1.244% | 0.369% |
| *Methanomethylovorans* | 0.170% | 0.085% | 0.189% | 0.967% | 0.625% | 0.058% | 0.359% | 0.077% | 1.068% | 0.400% | 0.371% |
| *Acinetobacter* | 9.668% | 22.159% | 5.266% | 10.342% | 15.671% | 15.247% | 13.682% | 11.326% | 10.479% | 12.649% | 4.504% |
| *Zoogloea* | 16.208% | 1.397% | 2.079% | 1.488% | 1.340% | 2.932% | 1.359% | 1.386% | 2.827% | 3.446% | 4.552% |
| *Desulfobulbus* | 1.841% | 3.871% | 12.893% | 5.101% | 3.000% | 2.036% | 2.888% | 3.315% | 5.222% | 4.463% | 3.182% |
| *Comamonas* | 0.671% | 0.603% | 0.321% | 7.937% | 0.753% | 4.145% | 1.030% | 1.088% | 0.534% | 1.898% | 2.398% |
| *Hyphomicrobium* | 0.447% | 0.567% | 0.499% | 0.362% | 0.625% | 0.562% | 0.504% | 0.553% | 0.367% | 0.498% | 0.086% |
| *Desulfosporosinus* | 0.008% | 0.000% | 0.000% | 0.000% | 0.000% | 0.005% | 0.000% | 0.000% | 0.005% | 0.002% | 0.003% |
| *Bacillus* | 1.877% | 0.378% | 0.321% | 0.340% | 0.356% | 0.419% | 0.507% | 0.499% | 0.542% | 0.582% | 0.464% |
| *Neisseria* | 0.027% | 1.638% | 0.795% | 0.756% | 0.049% | 2.600% | 1.132% | 5.899% | 0.101% | 1.444% | 1.763% |
| *Gracilibacteria* | 0.014% | 0.005% | 0.008% | 0.000% | 0.003% | 0.005% | 0.000% | 0.000% | 0.000% | 0.004% | 0.004% |
| *Rhodoferax* | 0.603% | 0.466% | 0.241% | 0.282% | 0.329% | 0.296% | 0.318% | 0.367% | 0.271% | 0.353% | 0.108% |
| *Streptococcus* | 0.652% | 4.077% | 1.827% | 0.537% | 0.784% | 1.312% | 2.718% | 3.822% | 4.197% | 2.214% | 1.434% |
| *Macellibacteroides* | 1.740% | 2.003% | 4.844% | 1.852% | 1.827% | 1.786% | 1.584% | 1.740% | 2.027% | 2.156% | 0.959% |
| *Christensenellaceae_R-7_group* | 3.436% | 3.244% | 5.121% | 2.844% | 3.142% | 2.819% | 2.523% | 1.973% | 2.526% | 3.070% | 0.836% |
| *Acidovorax* | 4.975% | 2.556% | 1.247% | 1.392% | 2.326% | 2.589% | 2.016% | 2.277% | 1.515% | 2.321% | 1.051% |
| *Thiothrix* | 0.047% | 0.041% | 0.011% | 0.019% | 0.055% | 0.022% | 0.016% | 0.033% | 0.049% | 0.033% | 0.015% |
| *Sphaerotilus* | 0.066% | 0.126% | 0.367% | 0.041% | 0.060% | 0.074% | 0.074% | 0.137% | 0.085% | 0.114% | 0.094% |
| *Salmonella* | 0.203% | 0.712% | 0.537% | 0.912% | 0.800% | 0.901% | 0.923% | 0.921% | 0.855% | 0.752% | 0.228% |
| *Hydrogenophaga* | 0.534% | 0.332% | 0.184% | 0.290% | 0.200% | 0.164% | 0.233% | 0.247% | 0.279% | 0.274% | 0.105% |

|  | **Treated wastewater** | | | | | | | | | | |
| --- | --- | --- | --- | --- | --- | --- | --- | --- | --- | --- | --- |
| **2 weeks** | **PP** | **PP** | **PP** | **PVC** | **PVC** | **PVC** | **HDPE** | **HDPE** | **HDPE** | **Mean** | **St.dev** |
| *Methylotenera* | 36.334% | 39.203% | 43.293% | 35.197% | 34.416% | 37.882% | 38.186% | 42.463% | 26.860% | 37.093% | 4.590% |
| *Methanomethylovorans* | 1.321% | 0.833% | 0.537% | 0.641% | 0.879% | 0.932% | 1.192% | 0.715% | 1.271% | 0.925% | 0.265% |
| *Acinetobacter* | 0.090% | 0.159% | 0.118% | 0.090% | 0.137% | 0.101% | 0.142% | 0.118% | 0.082% | 0.115% | 0.025% |
| *Zoogloea* | 0.685% | 0.529% | 0.488% | 0.570% | 0.556% | 0.625% | 0.523% | 0.436% | 0.685% | 0.566% | 0.080% |
| *Desulfobulbus* | 0.107% | 0.088% | 0.088% | 0.099% | 0.085% | 0.181% | 0.101% | 0.123% | 0.142% | 0.113% | 0.030% |
| *Comamonas* | 0.000% | 0.022% | 0.005% | 0.008% | 0.186% | 0.000% | 0.003% | 0.003% | 0.000% | 0.025% | 0.057% |
| *Hyphomicrobium* | 6.655% | 4.992% | 4.641% | 7.274% | 5.921% | 6.474% | 5.162% | 4.915% | 9.268% | 6.145% | 1.395% |
| *Desulfosporosinus* | 1.866% | 1.915% | 1.227% | 1.874% | 1.422% | 2.033% | 1.655% | 1.630% | 3.019% | 1.849% | 0.479% |
| *Bacillus* | 0.216% | 0.003% | 0.000% | 0.005% | 0.003% | 0.000% | 0.003% | 0.000% | 0.003% | 0.026% | 0.067% |
| *Neisseria* | 0.000% | 0.000% | 0.000% | 0.000% | 0.000% | 0.000% | 0.000% | 0.000% | 0.000% | 0.000% | 0.000% |
| *Gracilibacteria* | 1.496% | 3.575% | 5.115% | 2.575% | 3.326% | 2.715% | 4.408% | 5.652% | 1.395% | 3.362% | 1.408% |
| *Rhodoferax* | 4.814% | 4.986% | 4.504% | 5.244% | 4.416% | 4.301% | 4.970% | 4.266% | 5.575% | 4.786% | 0.425% |
| *Streptococcus* | 0.008% | 0.022% | 0.005% | 0.014% | 0.000% | 0.000% | 0.003% | 0.005% | 0.003% | 0.007% | 0.007% |
| *Macellibacteroides* | 0.016% | 0.025% | 0.022% | 0.022% | 0.027% | 0.014% | 0.025% | 0.019% | 0.025% | 0.022% | 0.004% |
| *Christensenellaceae_R-7_group* | 0.181% | 0.219% | 0.203% | 0.307% | 0.178% | 0.241% | 0.307% | 0.192% | 0.312% | 0.238% | 0.053% |
| *Acidovorax* | 0.995% | 1.016% | 1.074% | 1.025% | 0.923% | 1.093% | 1.063% | 0.997% | 1.071% | 1.029% | 0.050% |
| *Thiothrix* | 1.671% | 1.227% | 1.712% | 2.737% | 1.219% | 2.066% | 1.238% | 1.581% | 2.896% | 1.816% | 0.596% |
| *Sphaerotilus* | 3.003% | 3.844% | 2.778% | 3.499% | 3.351% | 3.066% | 3.447% | 3.074% | 4.762% | 3.425% | 0.560% |
| *Salmonella* | 0.247% | 0.027% | 0.036% | 0.047% | 0.203% | 0.036% | 0.041% | 0.030% | 0.071% | 0.082% | 0.078% |
| *Hydrogenophaga* | 2.521% | 2.778% | 2.860% | 2.797% | 2.556% | 2.638% | 2.701% | 2.608% | 3.381% | 2.760% | 0.244% |
| **4 weeks** | **PP** | **PP** | **PP** | **PVC** | **PVC** | **PVC** | **HDPE** | **HDPE** | **HDPE** | **Mean** | **St.dev** |
| *Methylotenera* | 25.942% | 22.077% | 23.378% | 19.816% | 20.477% | 22.956% | 28.660% | 21.433% | 18.605% | 22.594% | 2.953% |
| *Methanomethylovorans* | 9.195% | 9.734% | 14.307% | 11.940% | 13.562% | 5.918% | 7.326% | 17.781% | 26.353% | 12.902% | 5.885% |
| *Acinetobacter* | 0.096% | 0.082% | 0.088% | 0.088% | 0.052% | 0.126% | 0.066% | 0.066% | 0.058% | 0.080% | 0.022% |
| *Zoogloea* | 0.490% | 0.490% | 0.444% | 0.436% | 0.485% | 0.526% | 0.499% | 0.575% | 0.488% | 0.493% | 0.039% |
| *Desulfobulbus* | 0.482% | 0.534% | 0.589% | 0.932% | 0.641% | 0.762% | 0.589% | 0.452% | 0.542% | 0.614% | 0.141% |
| *Comamonas* | 0.019% | 0.041% | 0.008% | 0.044% | 0.030% | 0.019% | 0.030% | 0.005% | 0.003% | 0.022% | 0.014% |
| *Hyphomicrobium* | 4.263% | 4.321% | 3.118% | 3.748% | 3.510% | 4.184% | 5.162% | 5.485% | 5.058% | 4.316% | 0.750% |
| *Desulfosporosinus* | 6.307% | 6.534% | 6.260% | 5.893% | 7.205% | 6.962% | 5.447% | 6.156% | 5.326% | 6.232% | 0.590% |
| *Bacillus* | 0.063% | 0.052% | 0.000% | 0.041% | 0.044% | 0.041% | 0.063% | 0.003% | 0.000% | 0.034% | 0.025% |
| *Neisseria* | 0.000% | 0.000% | 0.000% | 0.000% | 0.000% | 0.000% | 0.000% | 0.000% | 0.000% | 0.000% | 0.000% |
| *Gracilibacteria* | 2.096% | 1.677% | 1.540% | 1.537% | 1.575% | 2.271% | 1.088% | 1.748% | 1.803% | 1.704% | 0.323% |
| *Rhodoferax* | 4.764% | 4.704% | 4.723% | 3.479% | 4.567% | 4.107% | 4.699% | 4.175% | 3.660% | 4.320% | 0.462% |
| *Streptococcus* | 0.011% | 0.014% | 0.049% | 0.019% | 0.019% | 0.005% | 0.016% | 0.003% | 0.008% | 0.016% | 0.013% |
| *Macellibacteroides* | 0.016% | 0.016% | 0.014% | 0.038% | 0.030% | 0.027% | 0.030% | 0.027% | 0.027% | 0.025% | 0.008% |
| *Christensenellaceae_R-7_group* | 0.688% | 0.759% | 0.611% | 1.068% | 0.833% | 0.671% | 0.658% | 0.666% | 0.740% | 0.744% | 0.130% |
| *Acidovorax* | 0.797% | 0.959% | 0.882% | 0.871% | 0.756% | 0.759% | 1.088% | 0.789% | 0.753% | 0.851% | 0.107% |
| *Thiothrix* | 3.463% | 4.885% | 4.488% | 3.942% | 3.603% | 4.378% | 3.104% | 1.641% | 3.526% | 3.670% | 0.896% |
| *Sphaerotilus* | 1.055% | 1.104% | 0.578% | 1.052% | 1.197% | 1.118% | 0.890% | 1.581% | 0.468% | 1.005% | 0.313% |
| *Salmonella* | 0.118% | 0.101% | 0.011% | 0.140% | 0.096% | 0.110% | 0.077% | 0.033% | 0.000% | 0.076% | 0.047% |
| *Hydrogenophaga* | 3.690% | 3.301% | 3.115% | 2.293% | 2.986% | 2.945% | 3.688% | 3.077% | 2.745% | 3.093% | 0.415% |
